# Promoting Polysulfide Redox Reactions through Electronic Spin Manipulation

**DOI:** 10.1021/acsnano.4c05278

**Published:** 2024-07-09

**Authors:** Jing Yu, Chen Huang, Oleg Usoltsev, Ashley P. Black, Kapil Gupta, Maria Chiara Spadaro, Ivan Pinto-Huguet, Marc Botifoll, Canhuang Li, Javier Herrero-Martín, Jinyuan Zhou, Alexandre Ponrouch, Ruirui Zhao, Lluís Balcells, Chao Yue Zhang, Andreu Cabot, Jordi Arbiol

**Affiliations:** † Catalan Institute of Nanoscience and Nanotechnology (ICN2), CSIC and BIST, Campus UAB, 08193 Bellaterra, Barcelona, Catalonia, Spain; ‡ Catalonia Institute for Energy Research (IREC), Sant Adrià de Besòs, Barcelona, 08930 Catalonia, Spain; § Department of Chemistry, University of Barcelona, 08028 Barcelona, Catalonia, Spain; ∥ ALBA Synchrotron, 08290 Cerdanyola del Vallès, Barcelona, Catalonia, Spain; ⊥ Institut de Ciència de Materials de Barcelona (ICMAB-CSIC), Campus de la UAB, 08193 Bellaterra, Barcelona, Catalonia, Spain; # Department of Physics and Astronomy “Ettore Majorana”, University of Catania, via S. Sofia 64, 95123 Catania, Italy; ¶ CNR-IMM, via S. Sofia 64, 95123 Catania, Italy; ∇ School of Chemistry, South China Normal University, 510006 Guangzhou, China; ○ Key Laboratory for Magnetism and Magnetic Materials of the Ministry of Education & School of Physical Science & Technology, Lanzhou University, 730000 Lanzhou, China; ⧫ ICREA, Passeig Lluìs Companys 23, 08010 Barcelona, Catalonia, Spain

**Keywords:** lithium−sulfur battery, vacancy, spin polarization, cobalt selenide, lithium polysulfide

## Abstract

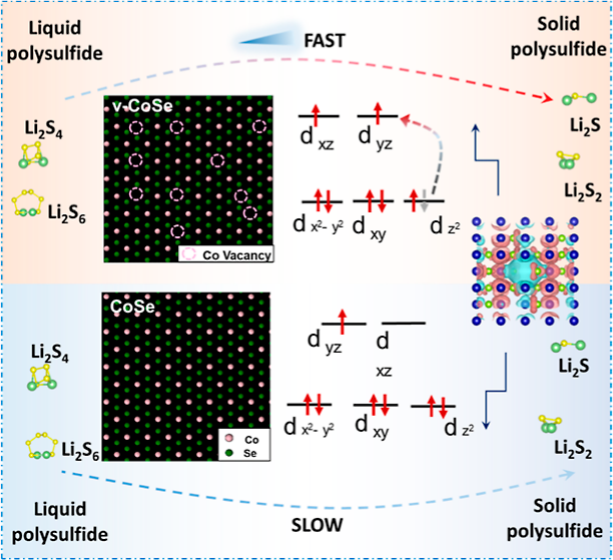

Catalytic additives able to accelerate the lithium–sulfur redox reaction are a key component of sulfur cathodes in lithium–sulfur batteries (LSBs). Their design focuses on optimizing the charge distribution within the energy spectra, which involves refinement of the distribution and occupancy of the electronic density of states. Herein, beyond charge distribution, we explore the role of the electronic spin configuration on the polysulfide adsorption properties and catalytic activity of the additive. We showcase the importance of this electronic parameter by generating spin polarization through a defect engineering approach based on the introduction of Co vacancies on the surface of CoSe nanosheets. We show vacancies change the electron spin state distribution, increasing the number of unpaired electrons with aligned spins. This local electronic rearrangement enhances the polysulfide adsorption, reducing the activation energy of the Li–S redox reactions. As a result, more uniform nucleation and growth of Li_2_S and an accelerated liquid–solid conversion in LSB cathodes are obtained. These translate into LSB cathodes exhibiting capacities up to 1089 mA h g^–1^ at 1 C with 0.017% average capacity loss after 1500 cycles, and up to 5.2 mA h cm^–2^, with 0.16% decay per cycle after 200 cycles in high sulfur loading cells.

## Introduction

Lithium–sulfur batteries (LSBs) are promising energy storage devices due to their high theoretical energy density (2600 W h kg^–1^), large specific capacity (1675 mA h g^–1^), potential cost-effectiveness, and environmental friendliness.^1^ However, the commercialization of LSBs is limited by factors such as polysulfide migration, sluggish kinetics of the sulfur redox reaction (SRR), and poor electronic and ionic cathode conductivities.^2^

While the introduction of porous carbon is crucial to promote mass and charge transport within the cathode, the use of catalytic additives is becoming essential to accelerate the SRR and thereby prevent sulfur loss through polysulfide migration. Notable research efforts have been devoted to developing catalysts able to promote the SRR kinetics.^3^ These catalytic materials have been tailored through the design and engineering of most of their parameters,^4^ including their composition,^5^ surface chemistry,^6,7^ architecture,^8^ interface,^9^ strain,^10^ and defects.^11^ Defect engineering, although not widely used in LSBs, is particularly straightforward and effective for adjusting the surface electronic structure of the catalyst. This adjustment allows for better control over the catalyst’s activity and selectivity, as shown in many other relevant electrochemical reactions.^12,13^ In particular, vacancies can expose unsaturated coordination bonds as active sites to enhance the adsorption of relevant species and activate their reaction.^14−16^ At the same time, atomic vacancies can introduce additional free charges and may even facilitate ionic transport.^17,18^ While numerous examples of the potential of defect engineering to promote the performance of several catalytic reactions have been reported, the precise manipulation of the defect type, location, electronic state, and concentration still poses a formidable challenge. Besides, the mechanisms behind the defect-related performance improvement are not well understood, which limits the rational design of defect-engineered catalysts, particularly in the field of LSBs.

Most previous work on defect engineering has focused on demonstrating that defects modify the electronic energy level position and occupancy, considering the carrier charge as the unique relevant parameter. Recently, the often overlooked electronic spin configuration of catalysts has emerged as a critical factor influencing their activity in electrocatalytic processes such as oxygen evolution.^19,20^ In the oxygen evolution reaction, the spin state strongly influences the catalyst’s electron transfer kinetics, the adsorption of intermediate species, and thus the overall activity.^21^ Various methodologies exist for manipulating the spin state of the catalyst. The simplest and most common one is the use of external magnetic fields to polarize the spins of ferromagnetic materials, which we have shown to strongly impact the performance of Li–S and Na–S batteries.^22,23^ Besides, external magnetic fields can also promote battery performance through magnetohydrodynamic effects.^24^ Despite the notable improvement in battery performance demonstrated in the lab, the application of external magnetic fields during battery operation is impractical in real-world applications. A more realistic solution to tune the electron spin state to promote the battery performance is the modification of the surface atomic coordination. Exchange interaction and spin states are closely intertwined with the atomic chemical environment which is determined by the crystal structure, atomic arrangement, and defects of the material. For instance, depending on the arrangement and coordination of d-orbital electrons, the spin state of transition metal cations, which is defined by the d-orbital electron spin configuration of the metal and its occupation, can vary between low-spin, intermediate-spin, and high-spin states. This spin state in turn determines the orbital interaction between the transition metal cation and the reactant.^25^

The coordination environment of surface active sites can be modified through atomic doping,^26,27^ the formation of heterojunctions,^28^ changing the geometric configuration,^29^ topographically disordered design,^30^ or adjusting the stoichiometry.^31,32^ In particular, beyond affecting the charge carrier density and thus the charge transport properties of the material, the introduction of vacancies modifies the spin coupling within the catalyst, thus the number of unpaired electrons. This is reflected by a change in magnetic properties related to the exchange interaction and crystal field effects, which determines the splitting of the d orbital energy level that can impact the catalyst activity and selectivity.^33−36^

Among the numerous SRR catalyst candidates, compared with other 3d transition metals, cobalt-based materials have generated particular interest due to their high electron density and large angular momentum of 3d electron orbit, enhancing its participation in electron transfer and the formation and breaking of chemical bonds.^36,37^ Thus, we selected CoSe as a catalytic material to showcase the effect of cation vacancies on the SRR activity. We analyze here how CoSe with cobalt vacancies undergoes spatial spin polarization while readjusting the surface electronic energy levels. This local spin rearrangement can activate the vacancy sites for effective chemical adsorption and conversion, which results in faster and more uniform nucleation and growth of the SRR solid products. As a consequence, the introduction of cation vacancies can significantly improve the electrochemical performance of LSBs. Ultimately, we evaluate here the electron spin state as a fundamental parameter to take into account when designing advanced SRR catalysts and how this spin state can be effectively controlled through defect engineering.

## Results and Discussion

### Vacancy Characterization

**Figure 1:**
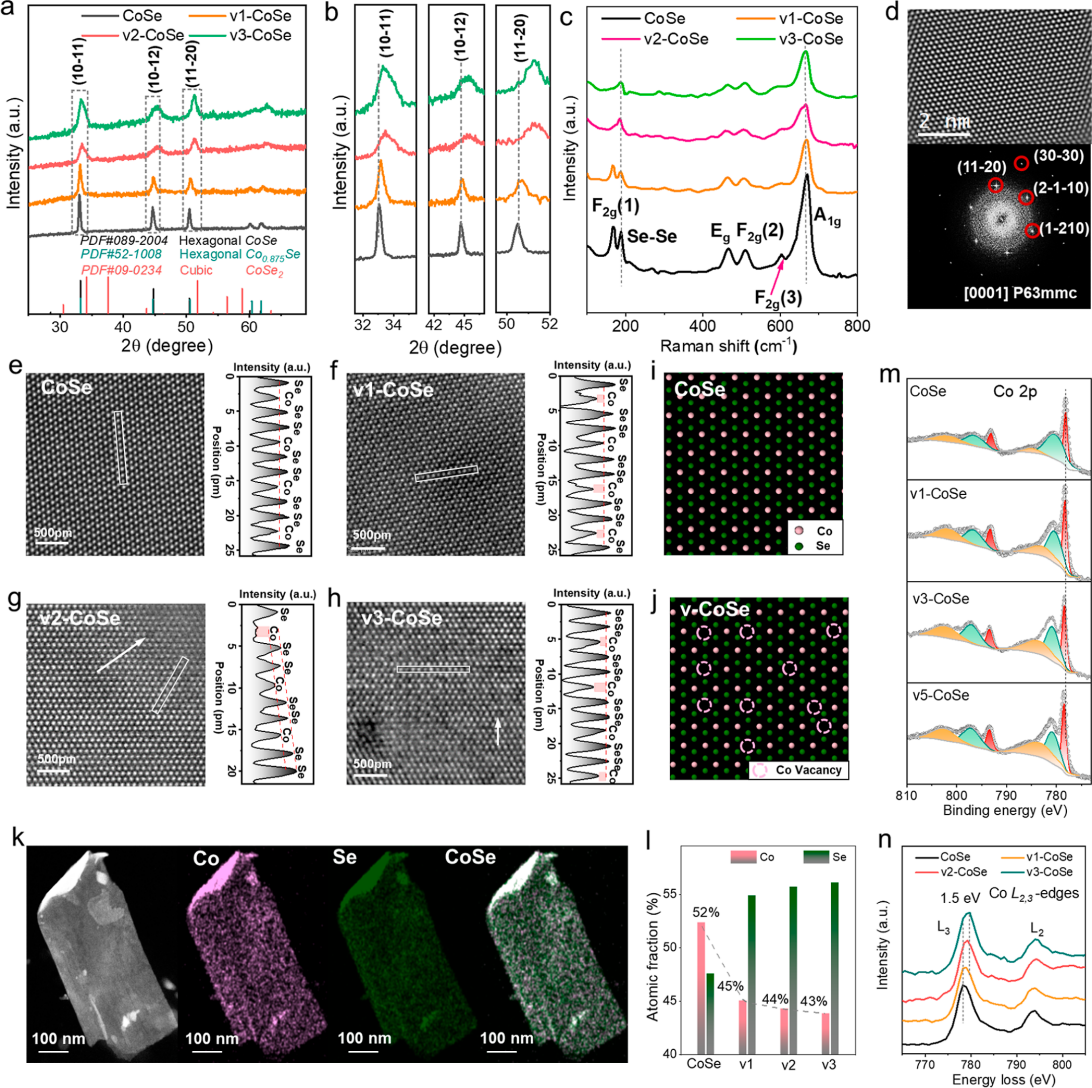
Characterization of vacancies in CoSe, v1-CoSe, v2-CoSe and v3-CoSe samples. (a,b) XRD patterns. (c) Raman spectra. (d) Atomic resolution AC-HAADF-STEM image of CoSe and corresponding indexed power spectrum FFT. The CoSe interplanar distances were measured to be 0.181, 0.180, and 0.103 nm, at 60.0 and 30.0°, which correspond to the (11-20), (2-1-10) and (30-30) lattice planes as visualized along its [0001] zone axis. (e–h) Atomic resolution AC-HAADF-STEM images with the intensity profiles along the selected rectangular regions suggesting missing Co atoms in (e) CoSe, (f) v1-CoSe, (g) v2-CoSe, and (h) v3-CoSe, respectively. (i,j) Atomic model schemes of the vacancies for (i) CoSe and (j) v-CoSe samples. (k) STEM-EDS elemental maps of v2-CoSe. (l) Co and Se atomic fraction obtained from STEM-EDS maps. (m) High-resolution Co 2p XPS spectra. (n) Co L-edge EELS spectra.

CoSe nanosheets extending several hundred nanometers and with a calculated thickness of 8.3 ± 1.6 nm (Figure S1) were produced by a facile hydrothermal synthesis approach (see details in the Experimental Section within the Supporting Information). Cobalt vacancies were generated using plasma etching. The amount was tuned by adjusting the etching time (see details in the Supporting Information). The plasma etched samples were generally named as v-CoSe, and more specifically as v1-CoSe, v2-CoSe, v3-CoSe, and v10-CoSe, according to the plasma etching time used (1, 2, 3, or 10 min, respectively). X-ray diffraction (XRD) patterns of all the samples (Figure 1a) display the fingerprint of the hexagonal CoSe phase.^38^ After extending the etching time to 10 min, the XRD pattern shows the presence of the CoSe_2_ phase along with the CoSe signal (Figure S2), which is consistent with a massive removal of Co. In contrast, v-CoSe samples that underwent a plasma etching time of 3 min or less do not show any additional peaks in the XRD pattern. Figure 1b displays the enlarged view of the main CoSe XRD peaks, showing a significant shift to higher 2θ values with the increase of etching time, corresponding to a reduction of the interplanar spacing. This reduction in the lattice parameters reflects the contraction of the hexagonal CoSe lattice with the introduction of Co vacancies, which favors a partial transition to Co_0.875_Se, and eventually to the cubic CoSe_2_ lattice (Figure S3). Besides, as the density of vacancies increased, a more disordered crystal was generated, as observed in the increase of the full width at half-maximum (fwhm) of the XRD peaks.^39^ While we aimed at introducing a controlled density of vacancies to study their effect as potential active reaction sites,^40^ because excessive plasma etching resulted in a rearrangement of the remaining cations and anions to form a different phase, the cubic CoSe_2_ phase, the v10-CoSe was not further considered in this study.

The characterization of the molecular bond vibrations by Raman spectroscopy was used to gain additional insight from the CoSe vacancies (Figure 1c). The sharp Raman peak at 187 cm^–1^ is related to the Se–Se bond vibration within CoSe.^41^ Besides, the signals at 167, 463, 510, 604, and 661 cm^–1^ are attributed to the CoSe F_2g_(1), E_g_, F_2g_(2), F_2g_(3), and A_1g_ modes, respectively.^42^ The intensity of the CoSe A_1g_ at 661 cm^–1^ decreases with the plasma treatment time, which is associated with an increase in the cation disorder. Besides, the intensity of the F_2g_(1) peak at 167 cm^–1^ decreased significantly to quasi-disappear, confirming the severe disorder of the Co ions within the v-CoSe lattice due to the large vacancy concentrations.

Atomic resolution aberration-corrected high-angle annular dark-field scanning transmission electron microscopy (AC-HAADF-STEM) was used to gain in-depth insights into the spatial distribution of Co vacancies. The AC-HAADF-STEM images together with their corresponding fast Fourier transform (FFT) patterns obtained from the nanostructures in Figures 1d and S4 reveal the CoSe and v-CoSe nanosheets to have the hexagonal crystal structure (space group = *P*63*mmc*) with *a* = *b* = 3.62(0) Å, *c* = 5.20(0) Å, which is consistent with XRD results. FFTs clearly show the (11-20), (2-1-10), and (1-210) lattice planes along the [0001] zone axis, which defines the nanosheet basal plane. The (11-20) lattice plane spacing of v-CoSe was measured at 0.179 nm, slightly shorter than the 0.181 nm obtained for pristine CoSe, in good agreement with XRD patterns.

Co vacancies are visible as relatively low-intensity white spots in the atomic resolution AC-HAADF-STEM images of the v-CoSe nanosheets, as shown in Figure 1e–h and represented in Figure 1i,j models. The intensity profiles along the selected rectangular regions in Figure 1e–h show the atomic columns of Co and Se to have some weaker Co signals pointing at a lower amount of cobalt atoms within the column, i.e., to the presence of Co vacancies. Besides the observable vacancies, additional line and plane defects can be found in the v-CoSe samples, particularly in the v3-CoSe sample (Figure 1f–h). Atomic models for the CoSe and v-CoSe nanostructures and the corresponding linear-STEM image simulations are displayed in Figure S5. The simulation results fit well with the experimental data, further demonstrating the relatively low-intensity peaks to be associated with Co vacancies.

Scanning electron microscopy (SEM) images combined with energy-dispersive X-ray spectroscopy (EDS) elemental maps show the Co and Se atoms to be homogeneously distributed throughout the nanosheet (Figure S1). The EDS-AC-HAADF-STEM maps of individual nanosheets (Figures 1k and S6–S9) further verify the homogeneous Co and Se compositional distribution at the nanometer scale. With the increase of the plasma etching time, the atomic ratio of Co decreases from 52% in the pristine CoSe sample to 45% (v1-CoSe), 44% (v2-CoSe), and 43% (v3-CoSe), as shown in Figure 1l. The nonlinear decrease in Co concentration may be related to the increasingly higher amount of energy required to remove additional Co from the structure.^43^

Nitrogen adsorption/desorption isotherms show the surface area of the samples to slightly increase with the plasma etching process. As displayed in Figure S10, the Brunauer–Emmett–Teller (BET) surface area was 224 cm^2^ g^–1^ for CoSe and 242 cm^2^ g^–1^ after plasma etching (v2-CoSe). This slight increase in surface area can be associated with the generation of some microporosity in the nanosheets or the dispersion of some stacked CoSe nanosheets aided by the plasma etching process.

X-ray photoelectron spectroscopy (XPS) analysis was used to study the surface chemistry of CoSe with different cation vacancy structures. As shown in Figure 1m, the high-resolution Co 2p XPS spectrum of CoSe shows the characteristic asymmetric main peaks of Co at 778.20 eV (3p_3/2_) together with a satellite peak at 785.21 eV, matching well the reported values for CoSe.^44^ The Co 2p XPS spectrum of v-CoSe shows a significant binding energy blueshift with respect to the CoSe, with the Co 3p_3/2_ position at 778.49 eV (v3-CoSe). This shift is attributed to the increased electronegativity of the chemical environment of the Co atoms that remain in the v-CoSe. This leads to an increased sharing of electrons between the Co atoms and the adjacent Se ions, i.e., an increase of the Co-oxidation state, which involves a higher energy cost to extract additional electrons. We also noticed that the Co satellite peak intensity increased gradually with the etching time, i.e., with the density of vacancies, which is related to the spatial spin polarization arrangement of 3d electrons, as discussed below.^45^

The vacancy-tuned electronic structure was analyzed using electron energy-loss spectroscopy (EELS). The Co L-edge EELS spectra show two strong L_3_ and L_2_ white lines for Co, ascribed to the electronic transition from the spin–orbit split levels 2p_3/2_ and 2p_1/2_ to the unoccupied 3d states. The charge transfer occurring during the vacancy creation processes is reflected in the change in energy position and intensity ratio of the L_3_ and L_2_ white lines.^46^ Compared with pristine CoSe, the L_3_-line energy in v-CoSe increased from 778.35 to 779.95 eV (Figure 1n), while the peak position of the Se L-edge remained constant (Figure S12), indicating a change in the electronic environment around Co vacancies. Moreover, we observed a shift in the relative peak intensity of Co L_3_/L_2_ from 2.56 (CoSe) to 1.16 (v-CoSe), which points to a higher occupation of Co 3d states in v-CoSe as the white-line ratio decreases with d occupancy (*n*_*d*_) when *n*_*d*_ > 5.^47^

A preliminary assessment of the influence of vacancies on the catalytic ability of the materials toward the conversion of lithium polysulfides was obtained using symmetrical cells, i.e., using CoSe, v1-CoSe, v2-CoSe, or v3-CoSe catalysts in both electrodes (see the Experimental Section in the Supporting Information for details).^48^ Cyclic voltammetry (CV) tests were performed in the voltage range from −1.5 to 1.5 V, using 0.5 M Li_2_S_6_ and 1 M lithium bis(trifluoromethanesulfonyl)imide (LiTFSI) in a 1:1 volumetric mixture of 3-dioxolane (DOL) and 1,2-dimethoxyethane (DME) as electrolyte. As shown in Figure S13 and Table S4, the v2-CoSe electrode shows the largest charge and the smallest overpotential, i.e., the lowest redox reaction barrier for lithium polysulfide conversion. Thus, v2-CoSe was the v-CoSe material considered for additional studies, and in the following, it is referred to as just v-CoSe.

### Spin State Structure

**Figure 2:**
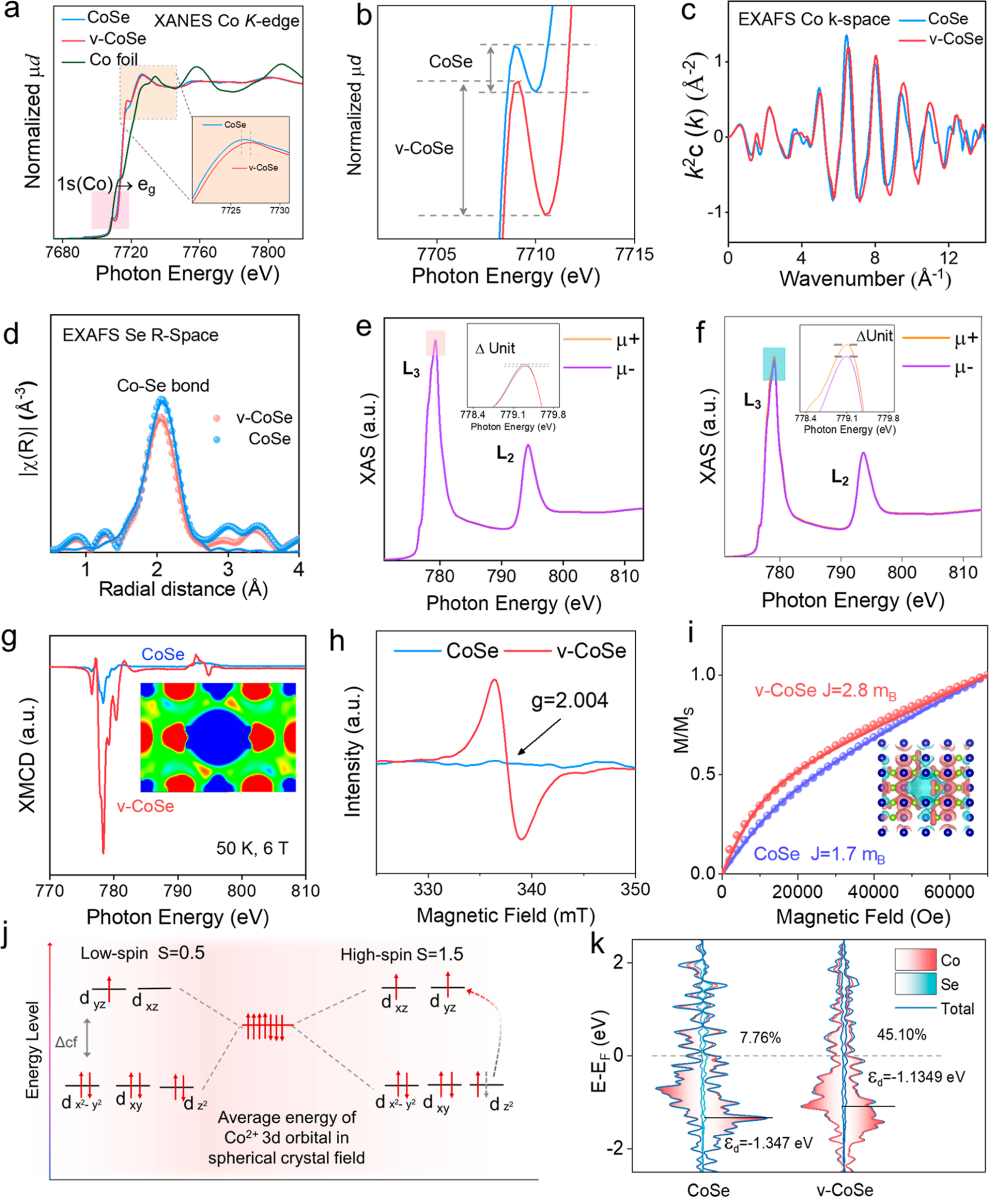
Spin configuration characterization of CoSe and v-CoSe. (a,b) Co K-edge XANES spectra. (c) EXAFS oscillation extracted from K-edge spectra of the composites in *k* space. (d) EXAFS fit of the composites. (e,f) Co L_2,3_ XAS (average of μ+ and μ−) of (e) CoSe and (f) v-CoSe at 50 K under an applied field *B* = 6 T. (g) XMCD (μ+ – μ−)/2 spectra. The inset shows the differential spin density between CoSe and v-CoSe. (h). EPR spectra. (i) Normalized magnetization curves at 2 K. The inset shows the differential spin density model between CoSe and v-CoSe. (j) Schematic diagram of electron spin–orbit configuration in Co^2+^. (k) Total density of states (TDOS) and partial density of states (PDOS).

The 3d orbital of Co^2+^ in CoSe splits in an octahedral field and it is very sensitive to the coordination environments. Extended X-ray absorption fine-structure spectra (EXAFS) and X-ray absorption near-edge structure spectroscopy (XANES) were used to analyze the electronic structures of Se and Co within CoSe and v-CoSe. In the Co K-edge spectrum, the 1s → 3d transition in the pre-edge region is particularly influenced by the spin state, oxidation state, and coordination geometry.^49^ As shown in Figure 2a, the Co pre-edge of both samples shows the conventional octahedral electron structure. A similar pre-edge peak was measured for CoSe and v-CoSe, involving no difference in the octahedron structure. The sharper peak at 7710.2 eV in v-CoSe (Figure 2b) is assigned to local electron transitions into unoccupied e_g_ states of the high-spin Co ion in an octahedral environment,^50,51^ which matches well with the satellite peaks observed in the Co 2p XPS spectra (Figure 1m). In terms of spin states, high-spin Co^2+^ usually has strong Co 2p satellite structures, about 5 eV higher in binding energy than the main Co 2p peak in the XPS spectra. On the other hand, low-spin Co^2+^ has associated a Co 2p XPS spectrum with less intense and poorly resolved satellite structures.^52,53^ The slight blueshift of the K-edge in XANES spectra of v-CoSe, as compared to CoSe, is related to a 20% increase in the average oxidation state of cobalt.^51^ In addition, the EXAFS oscillations obtained from the K-edge spectra show a lower amplitude in v-CoSe than in CoSe (Figures 2c and S14–S16), which is also related to the higher degree of disorder associated with the structural alteration in the Se coordination environment after extensive Co vacancy introduction.

The Fourier transformed k3-weighted Se K-edge EXAFS (Figure 2d) spectrum in the *R*-space of CoSe shows a peak at 2.4297 Å, corresponding to the Co–Se bond distance. For v-CoSe, the peak is shifted to lower distances, down to 2.4048 Å. The lower bond length is related to an increase in the Co-oxidation state in v-CoSe, and it is consistent with XRD, STEM EELS, Raman, and XPS results. Based on the EXAFS curve fits and derived structural parameters (Figure 2d), a lower coordination number for Se is obtained in v-CoSe (*N* = 4.2), compared to CoSe (*N* = 5.1), which is again consistent with the presence of a large density of Co vacancies. The Co Fourier transform-extended X-ray absorption fine structure (FT-EXAFS) results and the wavelet transform figures are in close alignment with those of Se. Overall, these data confirm that the introduction of Co vacancies results in a shorter Co–Se bond length, in agreement with the structural characterization results.

X-ray magnetic circular dichroism (XMCD) was used to gain information on the spin and orbital magnetic moment of Co and Se within CoSe and v-CoSe.^54^ Co L_2,3_ edges and Se L_1_ edge measurements were conducted at 50 K and 6 T. The XMCD spectra were obtained by subtracting the X-ray absorption spectroscopy (XAS) spectra acquired with photon helicity polarization antiparallel (σ^–^) and parallel (σ^+^) to the applied magnetic field, while the average XAS spectra were calculated as (σ^+^ + σ^–^)/2. As illustrated in Figures 2e–g, S17–S21, v-CoSe exhibits a strong Co dichroic signal with a spin magnetic moment significantly larger than that of CoSe according to the spin sum rule.^55^ This result probes the strong spatial spin polarization generated by the presence of Co vacancies on Co sites. On the other hand, no obvious changes were detected in Se L_1_ edge signals.

Both CoSe and v-CoSe exhibit paramagnetic behavior with antiferromagnetic correlations at low temperatures, Figure S22 shows the temperature dependence of the inverse magnetic susceptibility for v-CoSe at 50 Oe. The inverse susceptibility was fit in the paramagnetic range from 50 to 300 K to the Curie–Weiss law with some parasitic diamagnetic contribution. Figure 2i shows the magnetization curves at 2 K for CoSe and v-CoSe. The CoSe and v-CoSe curves were fitted with a linear susceptibility term that is probably related to antiferromagnetic interactions, and a Brillouin term that gives a moment per Co ion of approximately 1.7 and 2.8 μB, respectively. The increase in the effective moment for sample v-CoSe confirms the significant increase in the ratio of ions in the high spin state. The 7 electrons in the 3d orbit of Co^2+^ are preferentially distributed in those orbitals with lower energy according to the Hunter rule and the Pauli exclusion principle.^56^ Considering that each Co^2+^ ion can contribute either with 1 μB (low spin state) or 3 μB (high spin state), depending on their electronic configuration (Figure 2j), we estimate CoSe to have 35% of Co^2+^ ions in high spin state. This value increases to up to 90% in the v-CoSe sample. When considering the presence of 20% of Co^3+^ ions within v-CoSe and taking into account the three potential spin configurations (Figure S23), low spin (0 μB), medium spin (2 μB), and high spin (4 μB), we still estimate a percentage between 75 and 100% of Co^2+^ ions in the high spin state. The detailed calculation can be found in the Supporting Information. Overall, despite the uncertainty regarding the electronic arrangement of Co^3+^, v-CoSe demonstrates much higher spin polarization than CoSe.

The electron paramagnetic resonance (EPR) spectra of v-CoSe and CoSe are displayed in Figure 2h. The EPR spectrum of v-CoSe shows a strong signal at *g* = 2.004 not present in the CoSe sample, which further confirms the generation of additional electrons with unpaired spins by the introduction of Co vacancies (Figure 2i).^57^

To study the influence of cation vacancies on the electronic structure, the density of states (DOS) of the material was calculated by DFT (Figure S24). Despite the slight reduction in electron density resulting from the decrease in Co atoms, DFT calculation results show that the d band center of v-CoSe (−1.135 eV) is slightly closer to the Fermi level than that of CoSe (−1.347 eV). This shift involves an increase in orbital energy, thus electrons are more prone to disperse in the high-spin configuration. This result also indicates that the presence of vacancies in v-CoSe should enhance the material’s ability to adsorb relevant species during the catalytic process.^58^ We further differentiated between DOS with up and down electron spin states and determined the spatial spin polarization defined as^59^
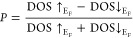
1where DOS↑_EF_ (or DOS↓_EF_) are the spin-up (spin-down) charge density calculated in the vacuum for an energy interval extending up to the Fermi level.^60^ According to this equation, the spatial spin polarization of CoSe and v-CoSe is 7.6 and 45.1%, respectively (Figure 2k).

The calculated charge and spin distribution in the surroundings of a Co vacancy and the differential maps between v-CoSe and CoSe in the surroundings are displayed in Figures S25 and 2g,i insets. We observe that the Co sites neighboring a Co vacancy show a redistribution of the charge density with higher electron densities in the vicinity of the vacancy site. Additionally, highly asymmetric spin distributions are generated in the vicinity of a Co vacancy. Such asymmetry and the higher charge density may facilitate electron transfer between v-CoSe and polysulfides and thus be helpful to the adsorption of polysulfides, as discussed below.

### Chemical Adsorption and Catalytic Mechanism

**Figure 3:**
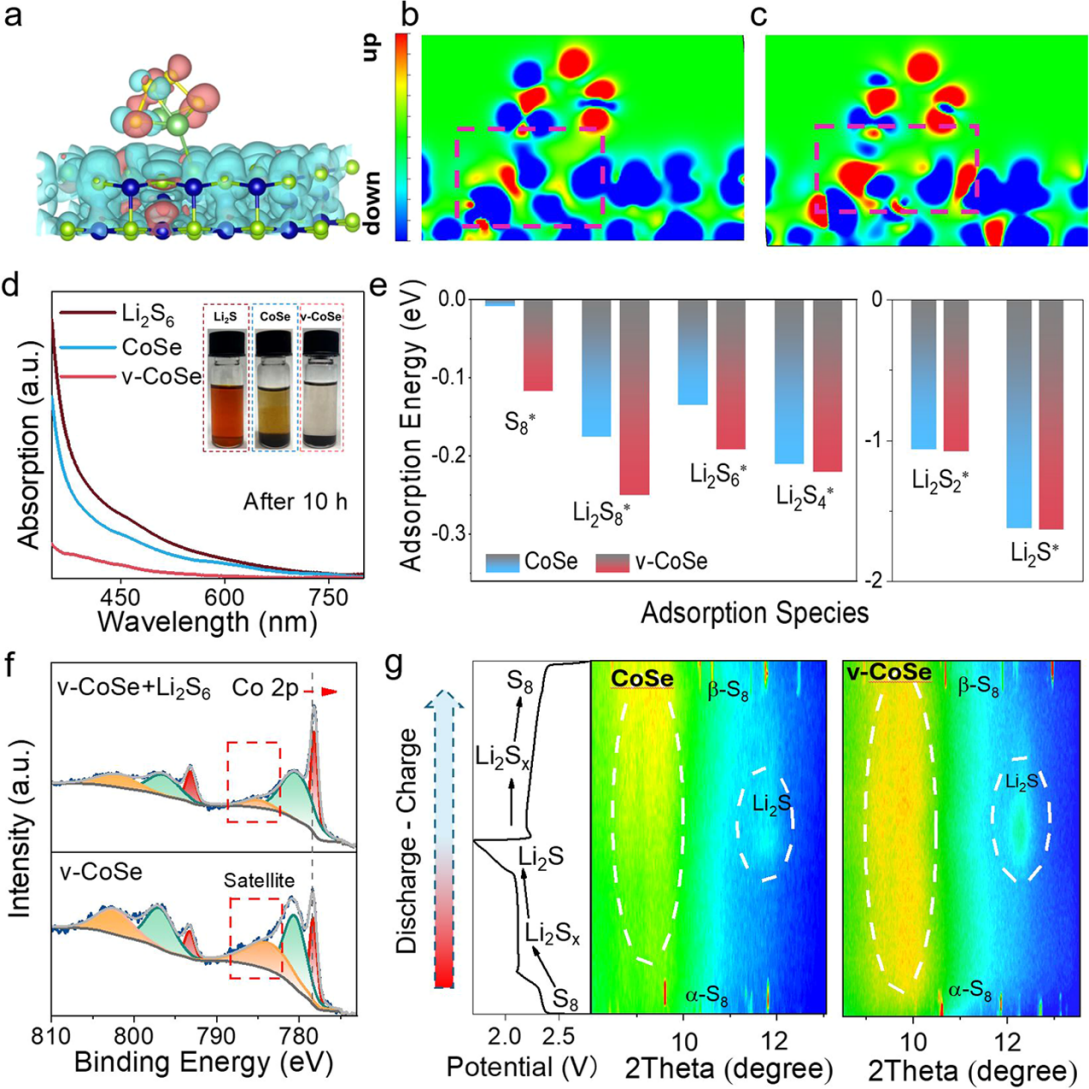
Adsorption characterization of CoSe and v-CoSe. (a) 3D spin density model of CoSe in conjunction with Li_2_S_4_, (b) 2D spin density map of CoSe and (c) v-CoSe with Li_2_S_4_. (d) UV–visible spectrum showing Li_2_S_6_ adsorption experiments, with optical photos inserted. (e) Adsorption energy of the surface for different sulfur species. (f) Co 2p XPS spectra obtained for v-CoSe before and after Li_2_S_6_ adsorption. (g) Operando XRD patterns during charging and discharging of batteries based on CoSe/S and v-CoSe/S.

To assess the difference in polysulfide adsorption capacity, DFT calculations were conducted. Figure 3a shows the model of the Li_2_S_4_ adsorbed on the catalyst surface and Figure 3b,c shows the spin state difference. A redistribution of the electron spins is observed, which illustrates the formation of a more stable adsorption bond. Experimentally, CoSe and v-CoSe were introduced in a Li_2_S_6_ solution (see details in the Supporting Information). After 10 h, the characteristic brown color of the dissolved Li_2_S_6_ molecules strongly decreased for the v-CoSe-containing solution, denoting major adsorption of the lithium polysulfide on the v-CoSe surface. On the other hand, the color of the solution containing CoSe showed a more moderate change, thus lower adsorption, as displayed in the optical photographs in the inset of Figure 3a. Ultraviolet–visible absorbance (UV–vis) spectra further confirmed this differential absorption in the visible and UV region of the spectra where Li_2_S_6_ shows a strong absorption band (Figure 3d).

DFT calculations were used to further investigate the interaction between CoSe and polysulfides. Figure 3e shows the adsorption energies of Li_2_S, the different polysulfides (Li_2_S_2_, Li_2_S_4_, Li_2_S_6_, Li_2_S_8_), and S_8_ on the surface of hexagonal CoSe, with and without Co vacancies. The surface adsorption configurations are shown in Figures S27–S30. Compared with CoSe, v-CoSe has significantly higher adsorption energies, thus improved adsorption ability, especially for soluble polysulfides (Li_2_S_4_, Li_2_S_6_, Li_2_S_8_) which are favor to the improvement of shuttle effect.^61^ This is related to the charge and spin redistribution near Co vacancies shown in Figure S25a–g. DFT calculations showed that after Li_2_S_6_ adsorption, the decrease in the electron spin density on the v-CoSe surface is accentuated when compared with CoSe, especially in the Se atom on the surface of v-CoSe (Figure S29). A similar result was obtained for the Li_2_S_4_ adsorption (Figure S30). Overall, the enhanced electrons transfer to the lithium polysulfide orbitals taking place in v-CoSe promotes their adsorption and facilitates subsequent catalytic reactions.

Experimentally, the Co 2p XPS spectrum of v-CoSe after the Li_2_S_6_ adsorption (Figure 3f) shows a 0.4 eV redshift. Additionally, upon adsorbing Li_2_S_6_, a significant decrease in the satellite peak of v-CoSe is observed, which indicates a decrease in the high spin electronic density related to the transference of electrons to the polysulfide.^62^ Both experimental results are in good agreement with DFT calculation outcomes.

LSB cathodes were prepared by loading S (69%, Figure S31) into the catalytic additive, CoSe or v-CoSe, and combining the mixture with super P and polyvinylidene fluoride (PVDF) binder in a ratio of 8:1:1. The material was coated on aluminum foil as the current collector, cut into circular pieces with a diameter of 12 mm. XRD patterns of the electrodes confirmed the presence of a notable amount of sulfur (Figure S32). For operando XRD experiments, coin cells with a 5 mm diameter window sealed with a Kapton were assembled using the CoSe/S or v-CoSe/S cathode, a Li anode, and a polypropylene separator. The electrolyte contained 1.0 M lithium bis(trifluoromethanesulfonyl) imide and 0.2 M LiNO_3_ in a DOL/DME (V/V = 1:1) mixture (see details in the Supporting Information). The operando X-ray powder diffraction patterns of the electrode materials during the first discharge and charge processes are illustrated in Figure 3g. In their pristine state, both CoSe/S and v-CoSe/S exhibit characteristic diffraction peaks corresponding to an orthorhombic α-S8 phase. Upon discharge, the sulfur signal diminishes rapidly, with no major diffraction peaks apparent until the end of discharge, where a weak signal corresponding to Li_2_S emerges, with the main peak at 12.5°. During the charging process, the Li_2_S signal decreases, and only at the final stage of charging the diffraction peaks corresponding to monoclinic β-S8 appear in both CoSe/S and v-CoSe/S. Additionally, subtle deformations in the background within the 8–10° region could be attributed to the formation of soluble polysulfides.^63^ This effect is particularly notable for v-CoSe/S, where the signal intensity increases notably at the end of discharge and the beginning of the charging process, reducing considerably by the end of charging, thus demonstrating high lithium polysulfide redox activity.

**Figure 4:**
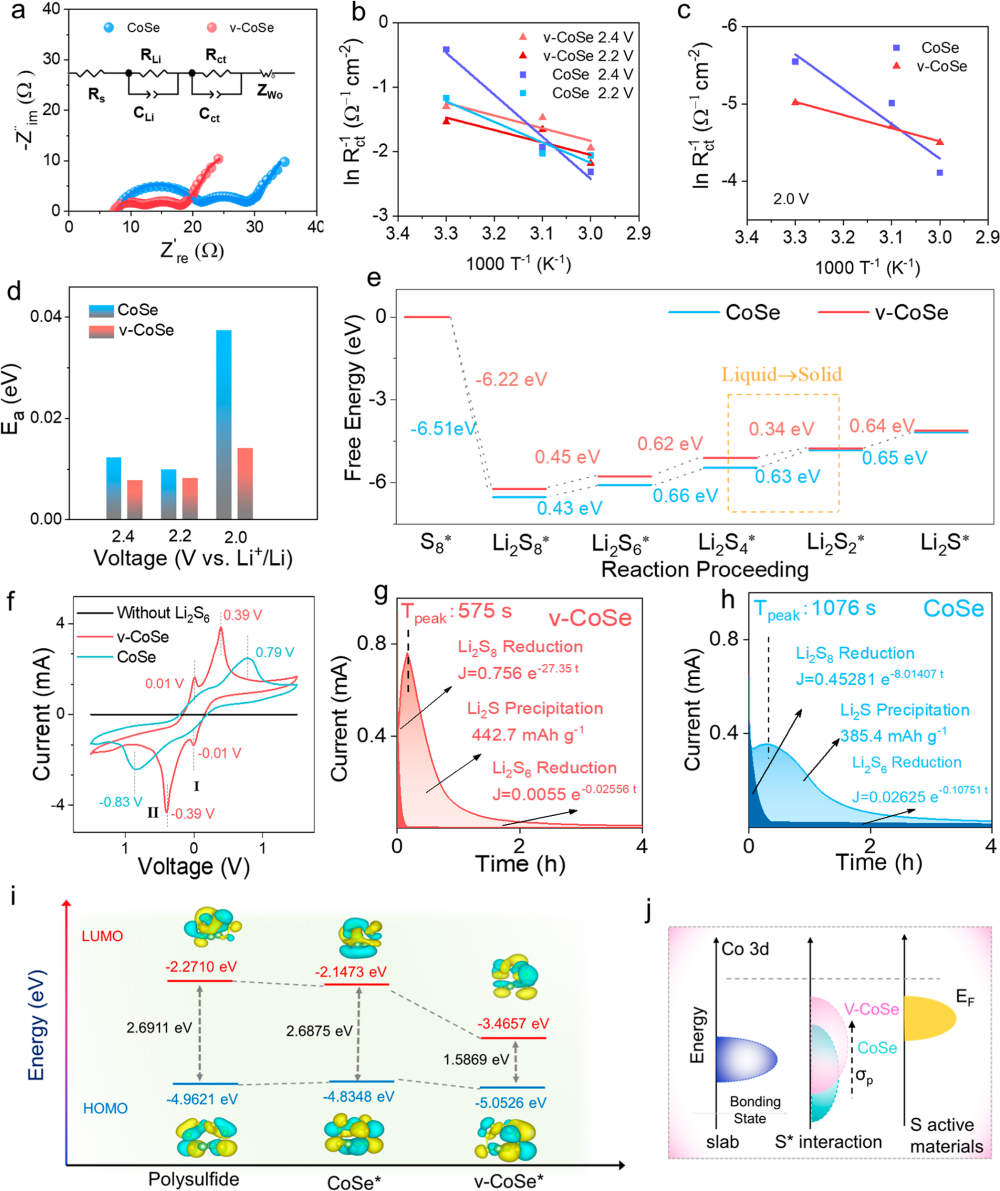
Catalytic characterization of CoSe and v-CoSe. (a) Fitted EIS spectra at 2.2 V and model circuit. (b,c) Arrhenius plot of *R*_ct_ calculated from CoSe (b) and v-CoSe (c). (d) Calculated activation energies at different voltages. (e) Gibbs free energy profiles and adsorption conformation of polysulfide species. (f) CV curves obtained from symmetric cells at a scan speed of 1 mV/s. (g,h) Potentiostatic discharge curves on CoSe (g) and v-CoSe (h) to study the Li_2_S nucleation kinetics. (i) Energy level scheme for pristine polysulfide and after adsorption. (j) Energy level diagram showing orbital hybridization for Li_2_S_4_ according to DOS results. *E*_F_ represents the Fermi level of the substrate; τ_p_ indicates bonding states.

The polysulfide conversion energy barrier during the discharging process was estimated using electrochemical impedance spectroscopy (EIS) measurements. In the discharge potential range between 2.4 and 2.0 V, the Nyquist plots of the EIS spectra show two semicircles. Considering the circuit model shown in the inset of Figure 4a, the intersection of the EIS spectra with the *x*-axis at high frequencies provides the internal resistance (*R*_s_) of the battery associated mainly with the electrolyte and electrical connection resistances. The higher frequency semicircle (*C*_Li_, *R*_Li_) is mainly related to the capacitance and charge transfer resistance of the Li/electrolyte interphase and includes also the contribution of the cathode porous structure.^64^ The lower frequency semicircle (*C*_CT_, *R*_CT_) is related to the charge transfer process at the cathode/electrolyte interphase that is associated with the polysulfide conversion process. The tail in the low-frequency range is related to the ion transfer resistance inherent to the cell, which is accommodated through a Warburg impedance term (*Z*_wo_).^65^ The activation energy (*E*_a_) of the charge transfer process linked to the polysulfide conversion was determined by fitting the temperature-dependence of the obtained charge transfer resistance (*R*_ct_) values using the Arrhenius eq (Figures S33 and S34)^66,67^
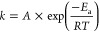
2
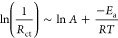
3where the reaction rate constant (*k*) is assimilated to the inverse of the charge transfer resistance (*R*_ct_), the pre-exponential factor (*A*) represents the collision frequency before reactants attain an excited state, and *T* is the absolute temperature. As shown in Figures 4b-d, throughout the voltage interval of 2.4–2.0 V, the SRR *E*_a_ of v-CoSe is significantly smaller than the values obtained from CoSe. In particular, the phase transition from liquid and soluble Li_2_S_*x*_ to solid and unsoluble Li_2_S_2_/Li_2_S plays an important role in the whole SRR process due to the substantial energy barrier associated with the nucleation and growth of the solid phase.^68^ Notably, at 2.4 and 2.2 V, corresponding to this liquid–solid conversion stage, there is a substantial reduction in activation energy with the introduction of the Co vacancies.

DFT calculation results (Figure 4e) considering the different spin states were consistent with the more favorable thermodynamic reduction process in v-CoSe compared with CoSe, especially during the liquid state (Li_2_S_4_) to solid-state Li_2_S_2_ transition, with a much lower energy barrier for v-CoSe (0.34 eV) than for CoSe (0.63 eV). The different energy is related to the spin-polarized electrons exhibiting enhanced transfer rates from the catalyst surface to the surface of liquid polysulfides (Li_2_S_6_ and Li_2_S_4_). This facilitates expedited solid–liquid conversion from Li_2_S_4_ to Li_2_S_2_, consequently optimizing solid-phase nucleation reaction kinetics for greater efficiency.

Symmetric cells were used to experimentally verify the above activation energy values and theoretical calculation results. CV curves acquired in the absence of Li_2_S_6_ in the electrolyte show very low currents (Figures 4f and S13), implying that the current response in the symmetric cell primarily arises from the conversion of the polysulfides within the electrolyte. In the presence of Li_2_S_6_, the freshly assembled symmetric cell shows an initial 0 V open-circuit voltage due to its electrode symmetry. However, as the negative scanning process progresses, Li_2_S_6_ gradually transforms into Li_2_S_4_ in one of the electrodes, as evidenced by peak I. Further reduction results in the conversion of Li_2_S_4_ to Li_2_S/Li_2_S_2_ (peak II). Both oxidation and reduction processes show cycling reversibility. Compared with the symmetric cell based on CoSe electrodes, the v-CoSe-based cell displays a lower polarization voltage and a sharper and higher current density at each oxidation–reduction peak. Specifically in peak II, v-CoSe shows a potential of −0.39 V, well below (in absolute value) that of CoSe, at −0.83 V. These experimental results underscore the outstanding catalytic efficacy of v-CoSe in facilitating the conversion of long-chain polysulfides, and are consistent with EIS spectra and DFT calculation results.

To gain deeper insight into the influence of vacancies on the nucleation and growth process, Li_2_S deposition experiments were conducted. In this experiment, the assembled coin cells were first galvanostatic discharged to 2.06 V, and then subjected to potentiostatic deposition at 2.05 V for 4 h. The shorter deposition time (575 s) observed from v-CoSe compared with CoSe (1076 s) indicates a much faster nucleation rate (Figure 4g,h). Applying Faraday’s law, the calculated Li_2_S deposition capacity for v-CoSe is 442.7 mA h g^–1^, well above that of CoSe at 385.4 mA h g^–1^, demonstrating an effective promotion of the Li_2_S nucleation process in v-CoSe. SEM images after deposition further supported these results, showing a larger amount of Li_2_S nucleated on the surface of v-CoSe than on the CoSe surface (Figure S35). This experimental result is consistent with the above calculation results showing that when the catalyst is combined with polysulfide, the active spin-polarized single electrons from v-CoSe are easier to transfer to the polysulfide to form bonds, achieving a rapid solid–liquid conversion process. This promoted charge transfer lowers the free energy required for nucleation, enhances polysulfide binding energy, and expedites surface electron exchange, thereby facilitating swift redox reactions.

DFT calculations were conducted to elucidate the electronic orbital occupation information on polysulfides after catalyst adsorption. The energy levels of the highest occupied molecular orbital (HOMO) and lowest unoccupied molecular orbital (LUMO) changes upon adsorption are shown in Figure 4i. The polysulfide adsorption on CoSe only marginally reduces its HOMO–LUMO band gap from 2.6911 to 2.6875 eV. On the other hand, the alteration in the spin configuration of Co in proximity to a Co vacancy results in additional unpaired electrons with a swift transferability from the catalyst surface to the polysulfide. This significantly diminishes the polysulfide LUMO upon adsorption and thus its HOMO–LUMO band gap is down to 1.5869 eV. This reduction in the band gap reflects the decrease of the polysulfide stability upon adsorption in the v-CoSe surface, thus enhancing the polysulfide conversion activity.

Figure 4j shows the energy level diagram of the orbital hybridization between Li_2_S_4_ and CoSe and v-CoSe surfaces. The calculated polysulfide DOS on the catalyst surface, especially at Co and vacancy sites, indicate that the polysulfide bonding on CoSe and v-CoSe has a different impact on the bonding states around the Fermi level of the sulfur atom^29,69^ (Figures 4j, S26 and S36), having the v-CoSe a higher density of electrons close to the *E*_F_. In brief, the adsorption of the polysulfide results in a notable shift of the bonding state toward the Fermi level, especially for v-CoSe.

### Electrochemical Performance

**Figure 5:**
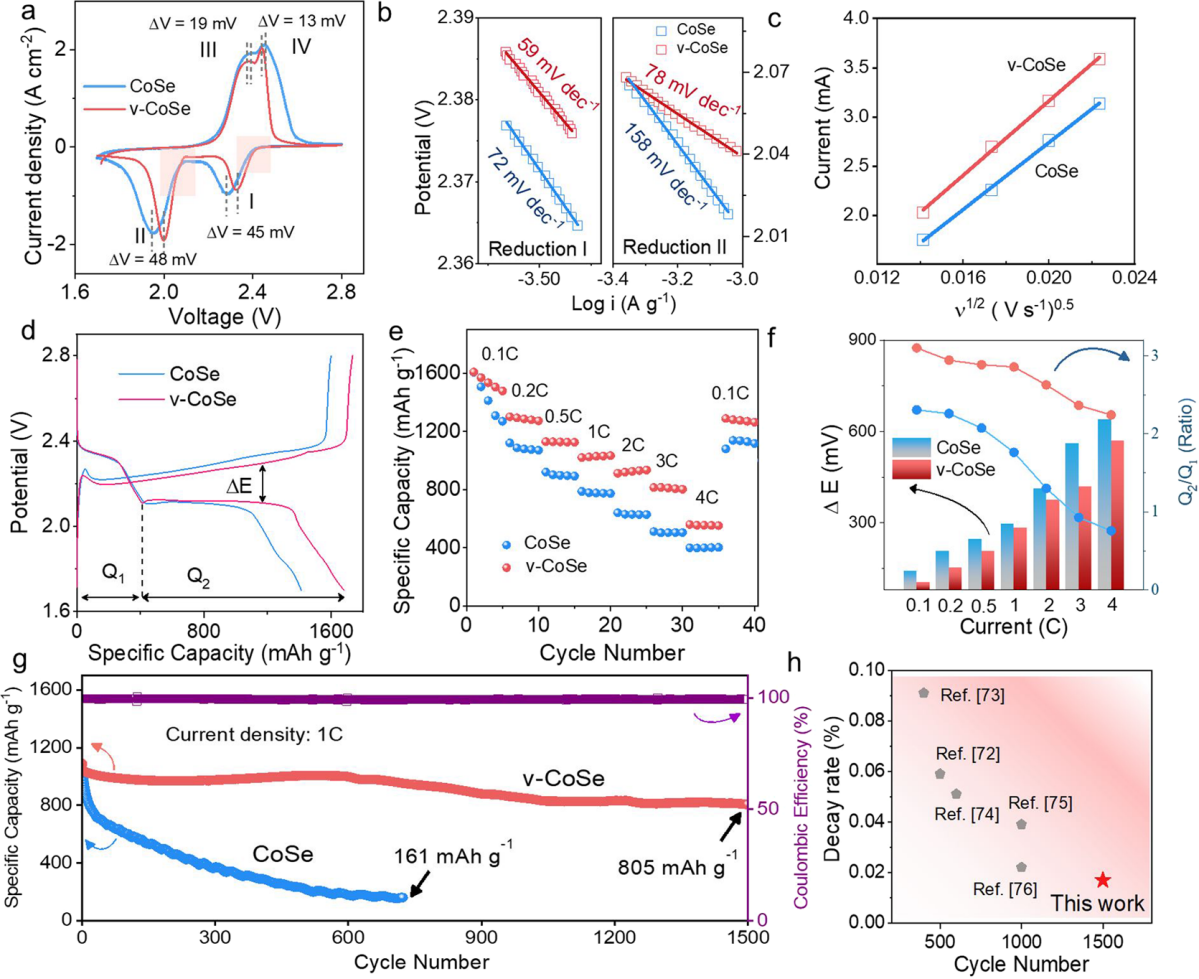
Electrochemical performance of v-CoSe/S and CoSe/S electrodes. (a) CV curves. (b) Tafel plots calculated from the reduction peaks I and II, respectively. (c) Peak current vs square root of scan rate in oxidation process. (d) GCD curves at 0.1 C. (e) Rate performance. (f) Δ*E* and *Q*_2_/*Q*_1_ values. (g) Long cycling performance at 1 C over 1500 cycles. (h) Comparison of the capacity obtained with the v-CoSe/S cathode with specific capacities of previously reported S catalysts electrodes.^72−76^

To evaluate the electrochemical performance of CoSe- and v-CoSe-based sulfur cathodes, CR2032 coin-type cells were assembled and cycled within the voltage range of 1.7–2.8 V, as detailed above and in the Supporting Information. Figure 5a displays the CV curve of a coin cell at a scan rate of 0.2 mV s^–1^, revealing several clear redox peaks. Notably, the v-CoSe electrode exhibited a stronger current response and sharper redox peaks. The reduction peak at 2.31 V accounts for the transformation from solid sulfur to long-chain polysulfide [Li_2_S_*x*_ (*x* ≥ 4)]. The subsequent reduction peak at 2.02 V denotes the liquid-to-solid conversion of long-chain polysulfides to Li_2_S_2_/Li_2_S. The oxidation peak represents the process of converting Li_2_S_2_/Li_2_S into long-chain polysulfides and ultimately into S_8_. The reduction peaks of v-CoSe/S exhibit a noticeable right shift when compared with those of CoSe/S, indicating a decrease in the polarization voltage during polysulfide conversion.

Figure S37 shows the linear sweep voltammetry (LSV) curve from the pink areas in Figure 5a,b shows the Tafel plots obtained from the reduction process in the LSV curves. The Tafel slope was calculated from a narrow voltage interval around the zero current voltage to prevent mass transfer limitation. The Tafel slope of the v-CoSe/S cathodic reduction process I was estimated at 59 mV dec^–1^, significantly below that of CoSe/S (72 mV dec^–1^). Notably, at the second reduction peak, v-CoSe/S displays a higher onset voltage and faster reduction current response signal compared to CoSe/S, yielding a Tafel slope of 78 mV dec^–1^ for v-CoSe/S, in contrast to CoSe/S with a Tafel slope of 158 mV dec ^–1^. This result further demonstrates the superior catalytic effect of v-CoSe/S on the polysulfide conversion process, particularly in the solid–liquid conversion of the second reduction process.

To compare catalytic reaction kinetics, CV curves of CoSe/S and v-CoSe/S at different rates (0.2 to 0.5 mV s^–1^) were measured (Figure S38). Each cathode exhibited a linear correlation between the peak current (*I*_p_) and the square root of the scan rate (*v*), indicating a diffusion-limited response (Figure 5c). According to the Randles-Sevcik equation, the Li^+^ diffusion coefficient (*D*_Li^+^_) can be calculated from the dependence of the peak current density versus the square root of the scan rate
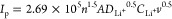
4

In this equation, *n*, *A*, and *C*_Li^+^_ represent the number of charges involved in the reaction, the electrode area, and the concentration of Li^+^, respectively. For CoSe/S and v-CoSe/S, the *D*_Li^+^_ estimated from the cathodic peaks are 3.78 × 10^–7^ cm^2^ s^–1^ and 4.96 × 10^–7^ cm^2^ s^–1^, respectively (Figure 5c).

The galvanostatic charge–discharge (GCD) curves of the two different cells at a current density of 0.1 C (1 C = 1675 mA h g^–1^) are shown in Figure 5d. The usual pair of discharge plateaus (*Q*_1_ and *Q*_2_) were measured for the two cells. v-CoSe/S shows a larger specific capacity, especially in the second discharge plateau (*Q*_2_). Besides, a smaller polarization voltage (Δ*E*), defined as the potential difference between the charge and discharge plateaus at 50% of the charge/discharge capacity, was measured for v-CoSe/S (38 mV) when compared with CoSe/S (52 mV). This result is consistent with the lower activation energy discussed above that was related to the fast liquid–solid conversion caused by the spin-polarized unpaired electrons transfer.

The rate performance of electrodes and the corresponding GCD at different currents are depicted in Figures 5e and S39. The clear discharge plateaus at rates up to 4 C indicate effective catalysis of the polysulfide conversion in the v-CoSe. With increasing current density, v-CoSe-based cells exhibit consistently higher initial specific capacities at all current densities compared with CoSe. In addition, upon returning to a current density of 0.1 C, v-CoSe/S demonstrates a substantial recovery of high specific capacity. Moreover, a lower Δ*E* is also observed at every current density, which further confirms the effective role of vacancy manipulation in promoting the Li–S reaction kinetics. As an example, at 0.1 C, v-CoSe/S exhibits a smaller polarization voltage of 103 mV compared to the 142 mV of CoSe/S (Figure 5f). Besides, the v-CoSe/S demonstrates higher *Q*_2_/*Q*_1_ values than CoSe/S consistently across all current densities presented in Figure 5f. As an example, the *Q*_2_/*Q*_1_ at 0.1 C was 2.88 for v-CoSe and just 2.30 for CoSe. *Q*_1_ accounts for the capacity contribution associated with the reaction of sulfur with Li^+^ to form soluble polysulfide.^70^*Q*_2_ accounts for the capacity contribution associated with the conversion of soluble polysulfide into solid Li_2_S/Li_2_S_2_. Hence, the ratio *Q*_2_/*Q*_1_ is an indicator of the polysulfide conversion efficiency. The higher *Q*_2_/*Q*_1_ value in v-CoSe/S than CoSe/S demonstrates a higher catalyst activity in the soluble polysulfide to solid Li_2_S/Li_2_S_2_ phase transformation.

The EIS spectra of the assembled coin cells during the discharge process and after 1500 charge and discharge cycles at a current density of 1 C are shown in Figure S40. The lower charge transfer resistance of v-CoSe/S at each discharge voltage compared with CoSe demonstrates a faster polysulfide conversion. Besides, the EIS fitting results after cycling reflect an excellent stability of the catalytic activity under continuous cycling.

The long-term cycling performance at a current density of 1 C is shown in Figure 5g. Both CoSe/S and v-CoSe/S electrodes have close initial specific capacities, 1089 mA h g^–1^ (v-CoSe) and 1047 mA h g^–1^ (CoSe), respectively. However, the capacity retention of v-CoSe/S is much higher, maintaining 805 mA h g^–1^ after 1500 cycles for just 161 mA h g^–1^ after 723 cycles in the CoSe/S electrode. The outstanding stability of v-CoSe/S is the result of the improved lithium polysulfide redox activity and low SRR activation energy, particularly for the liquid–solid conversion stage that promotes Li_2_S nucleation, and it is also manifested in the reduced polarization voltages and higher *Q*_2_/*Q*_1_ ratios. Besides, the v-CoSe/S electrode shows outstanding specific capacity retention compared with previously reported sulfur cathodes (Figure 5h). The analysis of the materials after cycling shows no major changes in the v-CoSe and CoSe structure, composition and chemical state of the elements (Figures S41–S46).

### High Sulfur Loading LSBs

**Figure 6:**
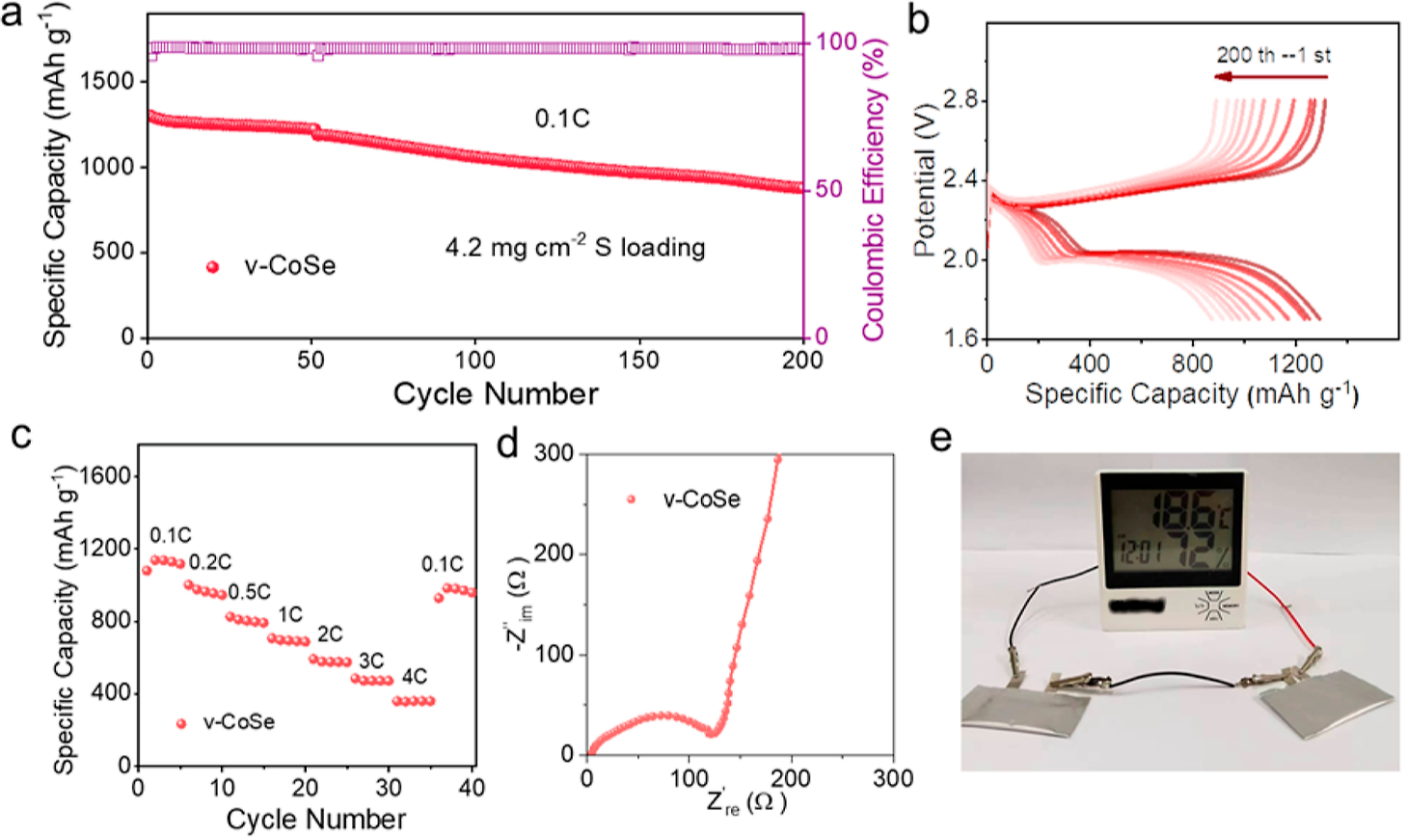
Electrochemical performance of v-CoSe/S at high sulfur loading. (a) Long-term cycling performance at 0.1 C. (b) GCD curves at 0.1 C from the 1st to the 200th cycles. (c) Rate performance from 0.1 to 4 C. (d) EIS before cycling. (f) Pouch cells powering a temperature and humidity monitoring clock.

To assess the practical application potential of LSBs based on v-CoSe/S electrodes, cells were assembled using larger sulfur loadings. Long-term cycling was conducted at a sulfur loading of 4.2 mg cm^–2^ and a current density of 0.1 C, as shown in Figure 6a. In these conditions, initial capacities up to 5.2 mA h cm^–2^, with only a 0.16% decay per cycle after 200 cycles, were measured. Additionally, the corresponding GCD curves reveal clearly defined plateaus, consistent with the excellent catalytic performance of v-CoSe (Figure 6b). Besides, as shown in the rate performance measurement (Figure 6c), v-CoSe demonstrates notable discharge-specific capacities at current densities up to 4 C. Moreover, the low *R*_ct_ (119 Ω) obtained from the EIS spectra (Figure 6d) indicates fast electron transfer even at this high sulfur loadings. Finally, Figure 6e showcases the capability of v-CoSe-based pouch cells to power our temperature and humidity monitoring clock, highlighting the practical utility of the fabricated electrodes.

## Conclusions

In this study, a defect engineering strategy was used to tune the electron spin state of an SRR electrocatalyst. This strategy was showcased by introducing controlled amounts of cation vacancies within ultrathin CoSe nanosheets. Theoretical calculations combined with experimental measurements showed the altered spin configuration results in enhancing both polysulfide adsorption and conversion activity and a more favorable thermodynamic reduction process, particularly for the transition from soluble polysulfide to solid Li_2_S that showed a lower nucleation energy barrier. v-CoSe cathodes not only demonstrated excellent electrocatalytic properties but also outstanding LSB electrochemical performance in terms of specific capacity, rate performance, and cycling stability, even at high sulfur loadings. Overall, this study demonstrates the need for considering the electronic spin configuration in the design of electrocatalysts, particularly for developing robust LSBs. Thus, the spin engineering approach showcased here facilitates the rational design of LSB cathodes based on defect-engineered SRR electrocatalysts, aiming toward the development of a cost-effective LSB technology with market-ready potential.

## Experimental Section

### Synthesis of CoSe

A facile hydrothermal synthesis approach was employed to produce CoSe nanosheets. Specifically, a solution containing 1 mmol of Co(AC)_2_·H_2_O and 1 mmol of Na_2_SeO_3_ in a mixed solvent (40 mL) with a volumetric ratio of ethylenediaminetetraacetic acid (EDTA)/deionized water set at 2:1 was prepared. Following 30 min of stirring, the resulting solution was transferred into a Teflon-lined autoclave, possessing a filling volume ratio of 80%. The autoclave was subsequently sealed and subjected to a thermal treatment at 180 °C for 16 h, followed by natural cooling to room temperature. The resultant black floccules were collected, subjected to washing procedures involving distilled water and absolute ethanol to eliminate residual ions and impurities, and subsequently dried under vacuum at 60 °C overnight.

### Synthesis of v-CoSe

Postdrying, plasma (radio frequency) etching was conducted on the synthesized material for a brief duration of irradiation time 1 min (v1-CoSe), power (400 W, 50 kHz continuously variable power supply) and pressure (60 Pa) with plasma rotating vacuum plasma instrument (TS-VPR10). Additional samples were obtained extending the plasma etching time to durations of 2, 3, and 10 min, denoted as v2-CoSe, v3-CoSe, and v10-CoSe, respectively.

### Synthesis of v-CoSe/S

The synthesis process of v-CoSe/S involves three sequential steps. First, the cathode host material, which comprises CoSe (or v-CoSe), is mixed with sulfur powder in a ratio of 1:3. Second, the mixture undergoes thorough grinding in a mortar and is subsequently subjected to 155 °C for 12 h. The resulting products, CoSe/S and v-CoSe/S, are then collected for further analysis.

### Synthesis of Li_2_S_6_ Solution and Adsorption Test

S and Li_2_S were dissolved into a mixed solution of 1,2-dimethoxyethane (DME) and 3-dioxolane (DOL) according to the ratio of 5:1, and then heated at 80 °C overnight to obtain the solution of Li_2_S_6_. To measure different sulfur cathode host materials, 15 mg host materials were soaked into the Li_2_S_6_ solution and stood for 10 h to observe the color change and measure their UV–vis absorption spectra.

## Supporting Information Available

Additional physical characterizations, electrochemical characterizations, and additional figures and tables (PDF)
