# Supplementary material for: Promoting Polysulfide Redox Reactions through Electronic Spin Manipulation
Source: ACS Nano. 2024 Jul 9;18(29):19268–82. doi: 10.1021/acsnano.4c05278 (PMC11271176; doi:10.1021/acsnano.4c05278)
Supplement: Supplementary file 1 — nn4c05278_si_001.pdf [file nn4c05278_si_001.pdf]

Supporting Information

Promoting Polysulfide Redox Reactions through Electronic Spin Manipulation

Jing Yu, Chen Huang, Oleg Usoltsev, Ashley P. Black, Kapil Gupta, Maria Chiara Spadaro, Ivan Pinto-Huguet, Marc Botifoll, Canhuang Li, Javier Herrero-Martín, Jinyuan Zhou, Alexandre Ponrouch, Ruirui Zhao, Lluís Balcells, Chao Yue Zhang, Andreu Cabot* and Jordi Arbiol**

J. Yu, K. Gupta, M. C. Spadaro, I. Pinto-Huguet, M. Botifoll and J. Arbiol
Catalan Institute of Nanoscience and Nanotechnology (ICN2), CSIC and BIST, Campus UAB, Bellaterra, 08193 Barcelona, Catalonia, Spain.
E-mail: arbiol@icrea.cat

J. Yu, C. Huang, C. Li and A. Cabot
Catalonia Institute for Energy Research (IREC), Sant Adrià de Besòs, 08930 Barcelona, Catalonia, Spain
E-mail: acabot@irec.cat

C. Huang, C. H. Li
Department of Chemistry
University of Barcelona 08028, Spain

O. Usoltsev and J. Herrero-Martín
ALBA Synchrotron,
Carrer de la Llum, 2, 26, 08290 Cerdanyola del Vallès, Barcelona, Catalonia, Spain

A. Black, A. Ponrouch and L. Balcells,
Institut de Ciència de Materials de Barcelona (ICMAB-CSIC), Campus de la UAB, 08193 Bellaterra, Catalonia, Spain

M. C. Spadaro
Department of Physics and Astronomy "Ettore Majorana", University of Catania, via S. Sofia 64, Catania 95123, Italy
CNR-IMM, via S. Sofia 64, Catania 95123, Italy

R. Zhao
School of Chemistry, South China Normal University, 510006 Guangzhou, China

C. Y. Zhang and J. Zhou
Key Laboratory for Magnetism and Magnetic Materials of the Ministry of Education & School of Physical Science & Technology, Lanzhou University, 730000 Lanzhou, China
E-mail: zhangchy2020@lzu.edu.cn

A. Cabot and J. Arbiol
ICREA, Pg. Lluis Company, 08010 Barcelona, Catalonia, Spain

** Corresponding authors*

Experimental Section

Symmetrical Cells Assembly and Measurements: Symmetrical cells were fabricated using a similar method as for lithium-sulfur batteries (LSBs). Two identical electrodes (CoSe or v-CoSe) were used as working and counter electrodes, respectively. 40 μL of 0.5 M Li_2S_6 and 1 M lithium bis(trifluoromethanesulfonyl)imide (LiTFSI) were dissolved in DOL/DME (V/V= 1:1) as the electrolyte. The loading mass of the electrode was about 0.5 mg cm^{-2}. The fabricated symmetric cells underwent testing using cyclic voltammetry (CV) at various scan rates.

Nucleation experimental test of Li_2S: The prepared sulfur cathode host material (CoSe and v-CoSe) was initially dissolved in an ethanol solution. Subsequently, it was coated onto carbon paper, serving as the working electrode, while lithium foil acted as the counter electrode. For the electrolyte, a solution consisting of 0.25 M Li_2S_8 with 1.0 M LiTFSI in tetraethylene glycol dimethyl ether solution was utilized as the catholyte, while a 1.0 M LiTFSI solution devoid of Li_2S_8 served as the anolyte. The assembled coin cells were firstly discharged to 2.06 V at a constant current and then subjected to potentiostatic deposition at a voltage of 2.05 V.

Material Characterizations: The crystal structure was examined using X-ray diffraction (XRD) with a D8 Advance instrument from Bruker. The morphology of the samples prepared in their

original state was analyzed through scanning electron microscopy (SEM), utilizing a field emission scanning electron microscope equipped with an electron column featuring a monochromator with UC (UniColore) Technology, specifically the FEI Magellan 400L. Atomic resolution aberration-corrected high angle annular dark field scanning transmission electron microscopy (AC-HAADF-STEM) was performed in a double aberration-corrected Thermo Fisher Spectra 300 STEM operated at 200 KeV. The Spectra 300 is equipped with a Super X energy dispersive X-ray spectroscopy (EDS) detector. 3D atomic models were obtained by using Rhodius and the corresponding linear-STEM image simulations using STEM-CELL software.[1-3] The surface composition and chemical state of the host materials were confirmed by X-ray photoelectron spectroscopy (XPS). Thermogravimetric analysis (TGA) was used to measure the S content in the cathode from 50°C to 400°C under a 5°C min^{-1} heating rate in an N_2 atmosphere. UV-vis absorption spectroscopy (Lambda 950 UV-Vis-NIR Spectrophotometer, Perkin Elmer) was used to analyze the adsorption performance of electrode materials on polysulfides. The X-ray absorption fine structure (XAFS) data were processed according to the standard procedures using the Athena module implemented in the IFEFFIT software package. The extended X-ray absorption fine structure (EXAFS) spectra were obtained by subtracting the post-edge background from the overall absorption and then normalizing with respect to the edge-jump step. Subsequently, the $\chi(k)$ data were Fourier transformed to real (R) space using Hanning windows (dk = 1.0 $Å^{-1}$) to separate the EXAFS contributions from different coordination shells. To obtain the quantitative structural parameters around central atoms, least-squares curve parameter fitting was performed using the ARTEMIS module of the IFEFFIT software packages. Magnetization measurements were done using a superconducting quantum interferometer device (SQUID, Quantum Design, from ICMAB's scientific and technical services). The temperature-dependent magnetization (M) measurements were carried out with a magnetic property measurement system superconducting

quantum interference device (MPMS SQUID) magnetometer and under magnetic field strength (*H*) of 50 Oe for all the samples.

Electrochemical Measurements: The prepared electrode material, Super P and polyvinylidene fluoride (PVDF) binder were mixed and ground according to 8:1:1, and N-methylpyrrolidone (NMP) was added during the grinding process to prepare slurry. Then it was evenly coated on the current collector on the aluminum foil, and finally dried at 60 °C for 12 h and cut into 12 mm circular pieces to obtain the working electrode. The sulfur loading content of the cathodes were around 1 mg cm^{-2}. To further highlight the practical application of the electrode, the high-loading cathode was prepared by the same method. The sulfur electrode, primed for electrochemical processes, served as the cathode, while lithium foil functioned as the anode, with Celgard 2400 serving as the separator, and 1.0 M LiTFSI and 0.1 M $LiNO_3$ were dissolved in DOL/DME (V: V=1:1) as the electrolyte to assemble a 2032-coin battery in the argon-filled glove box. The prepared battery is subjected to CV and GCD tests at a voltage window of 1.7 - 2.8V, and an EIS test is performed at a frequency of 0.1 Hz - 100K Hz.

Operando XRD: *Operando* XRD patterns were acquired on a Bruker D8 Advance A25 diffractometer in a Debye-Scherrer configuration equipped with a Mo Kα1 radiation source (λ = 0.7093 Å) using a costume holed-designed 2032 coin cells with a 5 mm diameter hole drilled and equipped with a 75 μm Kapton window. Diffraction patterns were measured upon continuous operation of the electrochemical cells between 8.5 and 13.5° with a step size of 0.02° and acquisition time of 60 min per pattern. The cells were cycled at C/25 rate with a Biologic SP-50 potentiostat in galvanostatic mode with potential limitation.

DFT calculations: All DFT calculations were performed using the Vienna ab-initio simulation package (VASP). The Perdew-Burke-Ernzerhof (PBE) functional for the exchange-correlation term was used with the projector augmented wave (PAW) potentials and a cutoff energy of 500 eV. The convergence of energy and forces were set to 1×10^{-5} eV and 0.05 eV/Å, respectively. The adsorption energy E_{ad} was calculated as:

$$E_{ad} = E_{(surf+ad)} - E_{surf} - E_{ad}$$

where $E_{(surf+ad)}$ is the energy of the LiPS adsorbed on the surface, E_{surf} is the energy of the clean surface, and E_{ad} is the energy of the free LiPS.

The formula for calculating the Gibbs free as fellow:

$$\Delta G(S_8\text{-}Li_2S_8) = E_{Li_2S_8} - E_{S_8} - 2E_{Li^+}$$

$$\Delta G(Li_2S_8\text{-}Li_2S_6) = E_{Li_2S_6} - E_{Li_2S_8} + 0.25E_{S_8}$$

$$\Delta G\ (Li_2S_6\text{-}Li_2S_4) = E_{Li_2S_4} - E_{Li_2S_6} + 0.25E_{S_8}$$

$$\Delta G(Li_2S_4\text{-}Li_2S_2) = E_{Li_2S_2} - E_{Li_2S_4} + 0.25E_{S_8}$$

$$\Delta G(Li_2S_2\text{-}Li_2S) = E_{Li_2S} - E_{Li_2S_2} + 0.125E_{S_8}$$

Pouch cell assembly and measurements: The v-CoSe /S cathode and lithium anode were cut into 4×3 cm pieces. The sulfur loading of the cathode in the pouch cell was 1.8 mg cm^{-2}. The E/S ratio was about 20 μL mg^{-1}, and the thickness of the lithium belt anode was 0.4 mm. The separator and electrolyte were sandwiched between the tailored v-CoSe /S and lithium belt.

Calculation of spin-state: We name the low spin ratio of Co^{2+}in CoSe as $R^{2+}{}_{low}$, and the high spin ratio of Co^{2+}in CoSe as $R^{2+}{}_{high}$, so we could perform a formula (1-2) below.

In CoSe: 1 muB * R^{2+}_{low} + 3 muB * R^{2+}_{high}=1.7 muB (1)

In v-CoSe: 1 muB * R^{2+}_{low} + 3 muB * R^{2+}_{high}=2.8 muB (2)

Thus the R^{2+}_{low} =65%, R^{2+}_{high} = 35% in CoSe from formula (1), while R^{2+}_{low} =10%, R^{2+}_{high} = 90% in v-CoSe from formula (2).

To be more specific, in the case of v-CoSe supposed to take into account the presence of 20% of Co^{3+} ions, which has the three potential spin configurations (Figure S23), low spin (0 muB), medium spin (2 muB), and high spin 4 (muB), then the calculation formula should apply the following formula (3):

80%*(1 muB * R^{2+}_{low} + 3 muB * R^{2+}_{high})+ 20%*(0 muB * R^{3+}_{low} + 2 muB * R^{3+}_{med}+ 4 muB * R^{3+}_{high})=2.8 muB (3)

In this scenario, since there are too many uncertain values, we assume two extreme cases which is if all Co^{3+} is with low spin(R^{3+}_{low}=1, R^{3+}_{med}=0, R^{3+}_{high}=0), then R^{2+}_{high}=100%; Or all Co^{3+} is with high spin, (R^{3+}_{low}=0, R^{3+}_{med}=0, R^{3+}_{high}=1), then R^{2+}_{high}=**75%**.

Thickness calculation:

To calculate the thickness of the nanosheets composing our sample, we utilize low-loss EELS spectra. The thickness (t) is computed using the logarithmic-ratio formula (3).

$$t = \lambda \ln(\frac{I_t}{I_0}) \qquad (3)$$

Where λ represents the inelastic mean free path, I_t denotes the total area under the low-loss EELS spectrum and I_0 is the area under the zero-loss peak. The logarithmic term is computed using DigitalMicrograph 3.0 software. We designate a specific region of the image and determine a value of 0.09 for this term.

To calculate the λ, we employ Equation (4) as proposed by T. Malis et al. [4]:

$$\lambda = \frac{106\, F\, E_0}{E_m \ln(\frac{2\beta E_0}{E_m})} \qquad (4)$$

Where E_0 is the incident energy in keV, β is the collection semiangle mrad, Z is the effective atomic mass of our material and:

$$F = \frac{1 + \frac{E_0}{1022}}{\left(1 + \frac{E_0}{511}\right)^2} \qquad (5)$$

$$E_m = 7.6\, Z^{0.36} \qquad (6)$$

The parameter values for our sample are presented in Table S1. Substituting these values into the equations yields $\lambda = 92$. Thus, utilizing Equation (3) and considering a 20% error in this calculation, we determine a thickness of 8.3 ± 1.6 nm.

Table S1. Values used for λ calculation.

$\boldsymbol{E_0}$	$\boldsymbol{\beta}$	$\boldsymbol{Z}$
200 keV	15.1 mrad	30.5

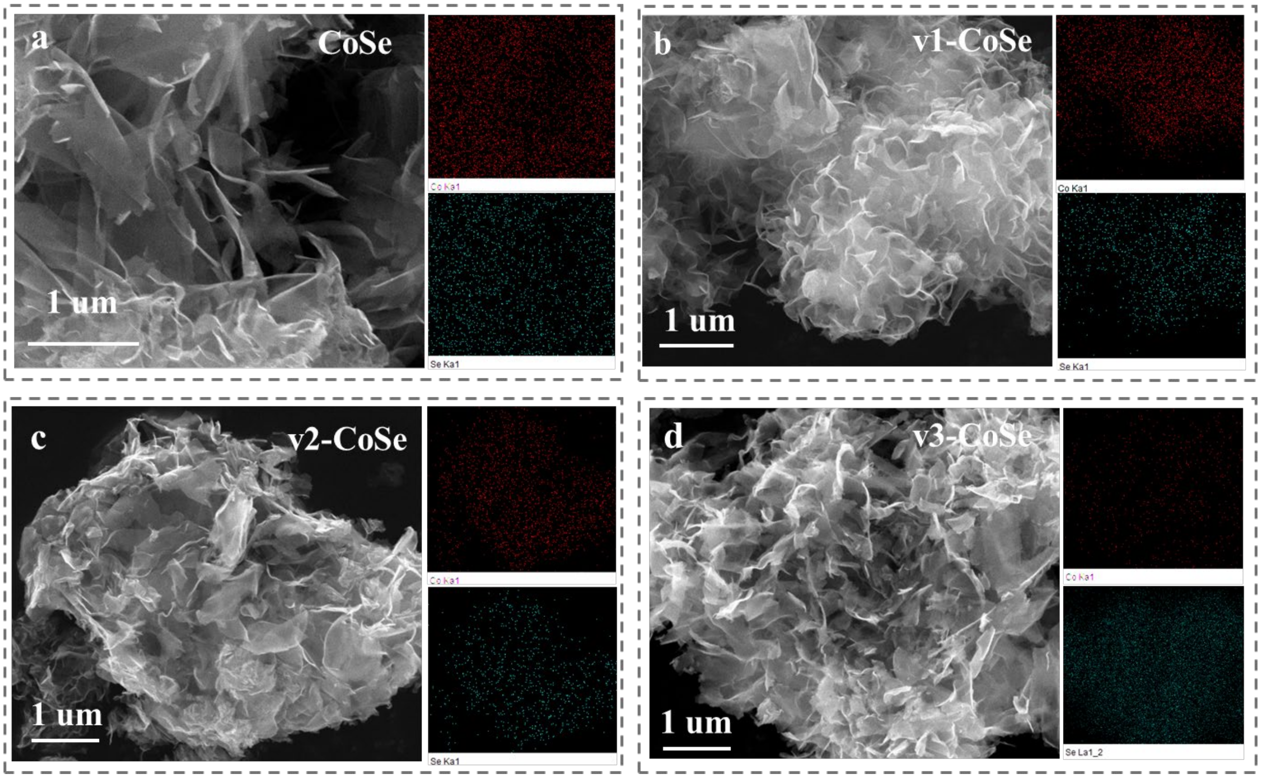

Figure S1. SEM images and EDS composition maps of (a) CoSe, (b) v1-CoSe, (c) v2-CoSe, (d) v3-CoSe. Red = Co; Blue = Se.

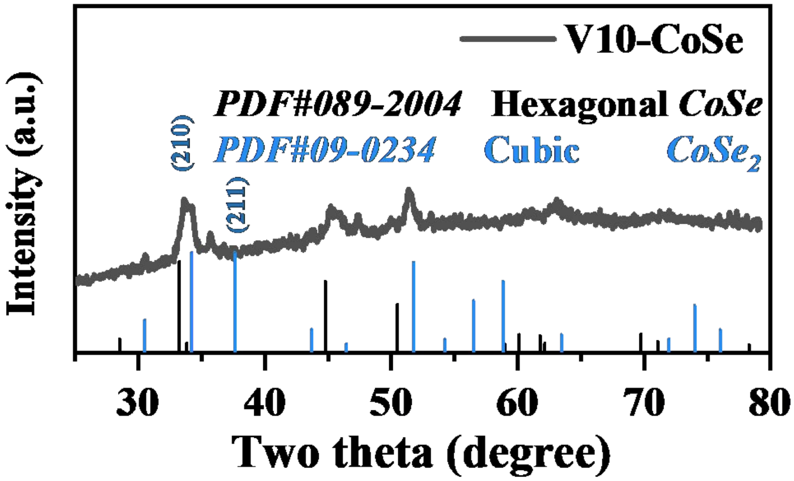

Figure S2. XRD patterns of v10-CoSe and hexagonal and cubic CoSe and $CoSe_2$ references. The unmatched peak intensities are related to the asymmetric geometry of the nanosheet.[5]

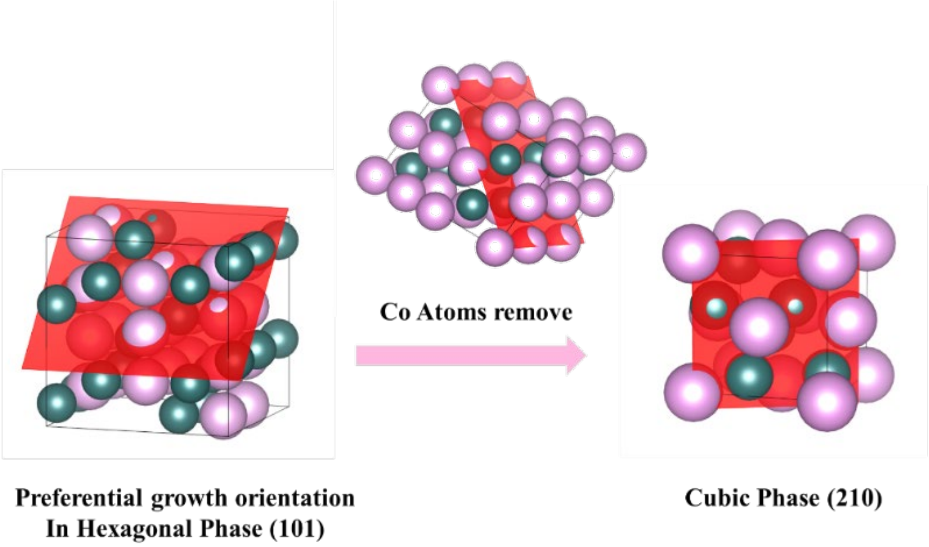

Figure S3. Shematic diagram of phase changes caused by the removal of excessive Co from hexagonal CoSe to cubic $CoSe_2$.

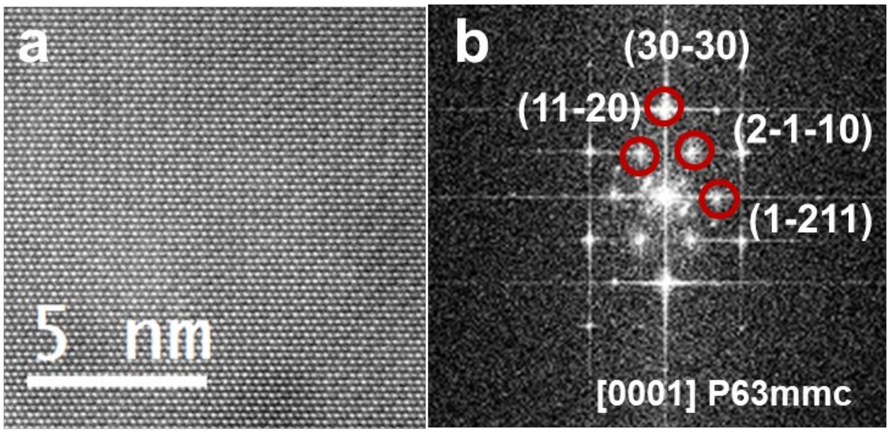

Figure S4. AC-HAADF-STEM image (a) and the corresponding FFT (b) of a v-CoSe nanosheet. The lattice fringe distances were measured to be 0.179 nm, 0.179 nm and 0.102 nm, at 60.0º and 30.0º, which correspond to the (11-20), (2-1-10) and (30-30) lattice planes as visualized along its [0001] zone axis.

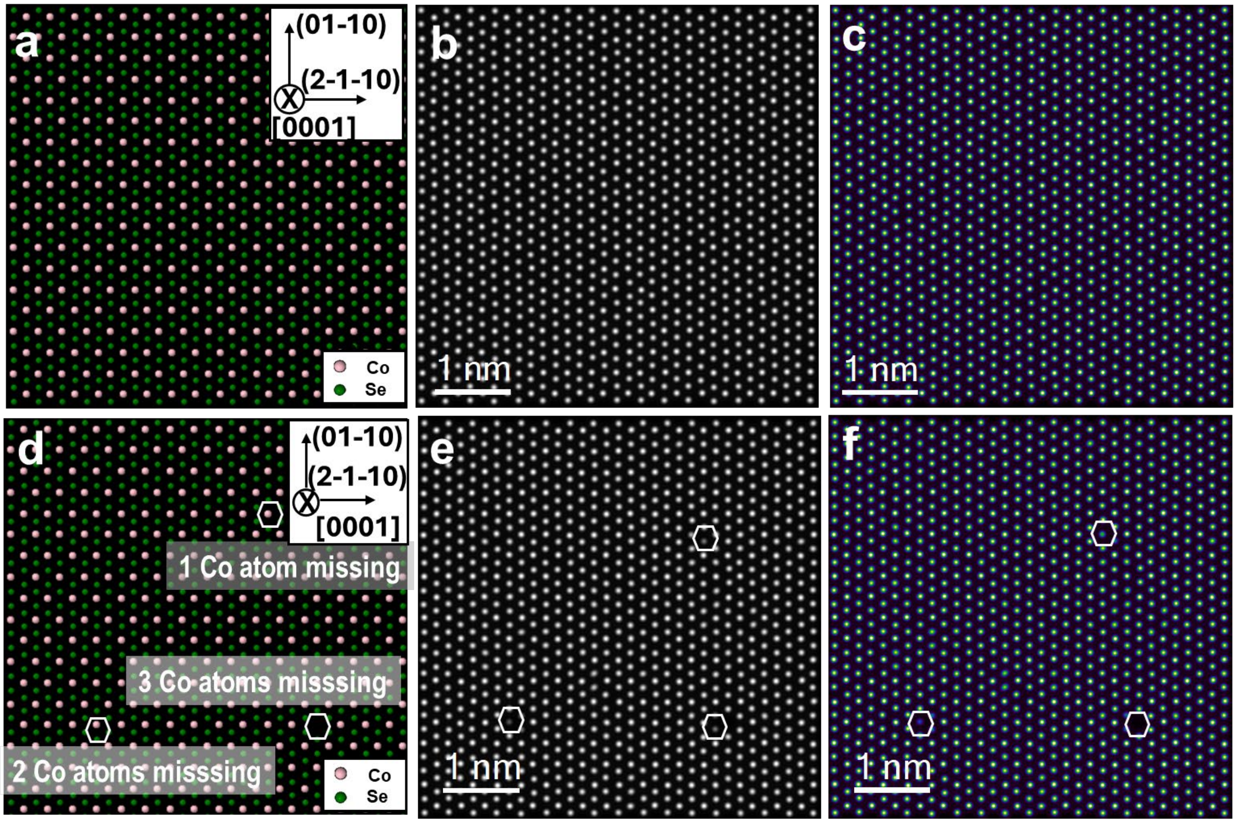

Figure S5. Atomic models and the corresponding simulated AC-HAADF-STEM images for (a-c) CoSe, and (e-f) v-CoSe along its [0001] zone axis, evidencing the different image contrast dependence on the number of Co atoms vacancy within the same atomic column.

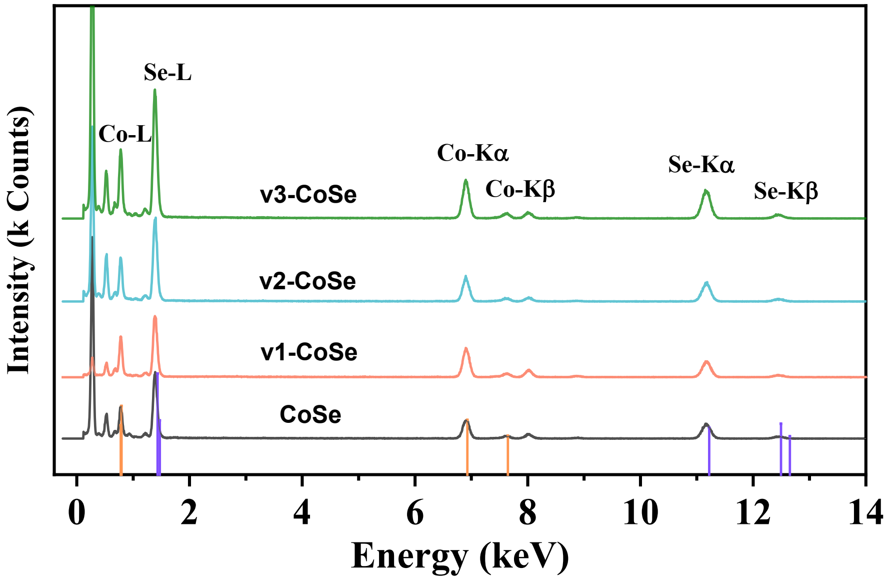

Figure S6. EDS spectra illustrating the elemental composition of CoSe and v-CoSe.

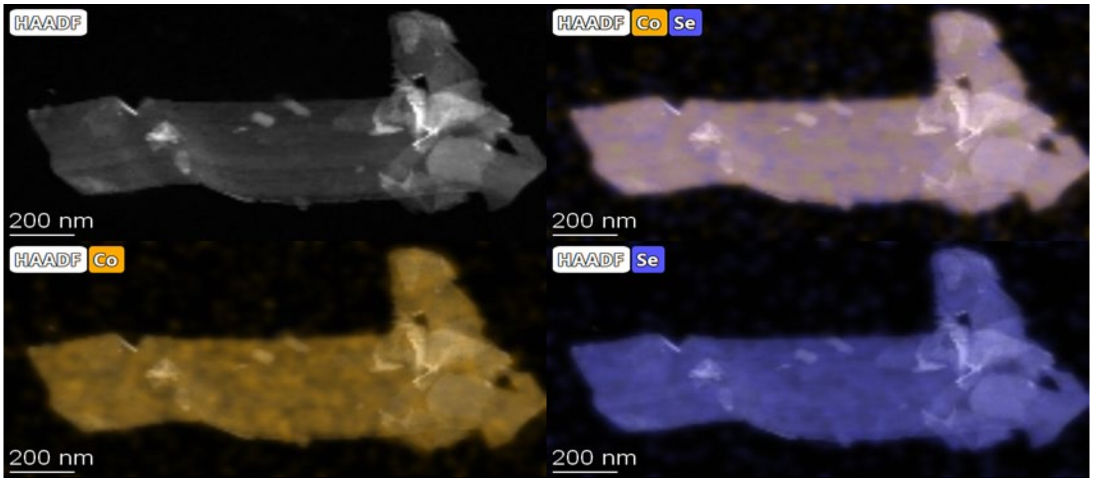

Figure S7. EDS elemental maps of CoSe.

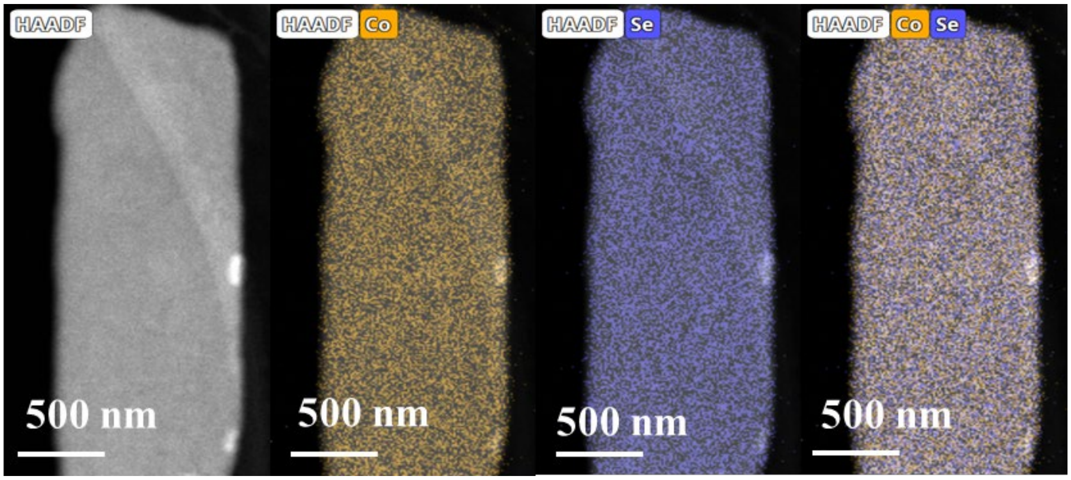

Figure S8. EDS elemental maps of v1-CoSe.

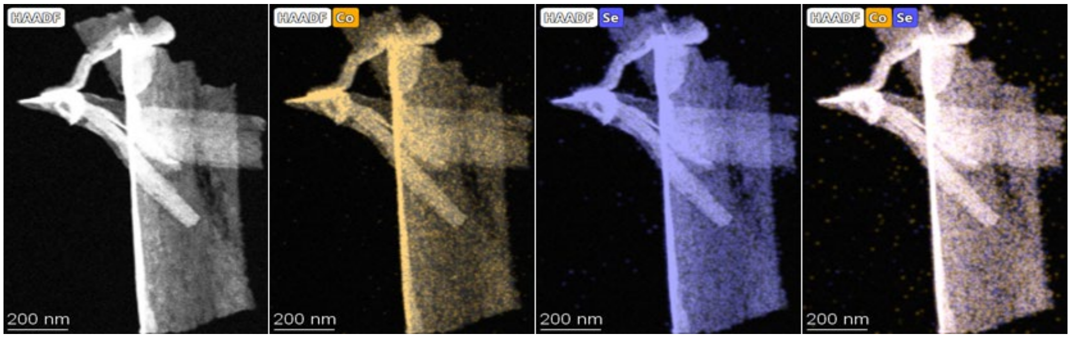

Figure S9. EDS elemental maps of v3-CoSe.

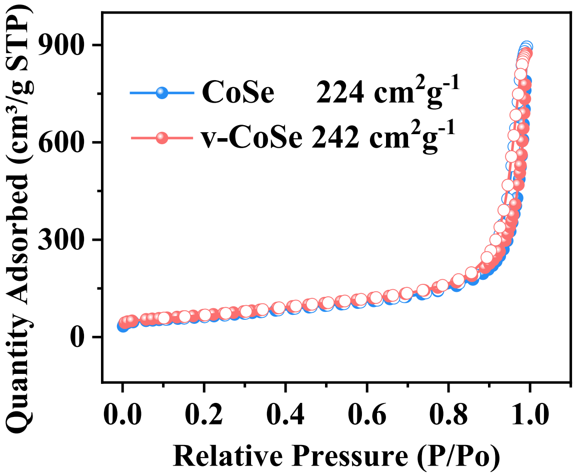

Figure S10. Nitrogen adsorption/desorption isotherms of CoSe and v-CoSe.

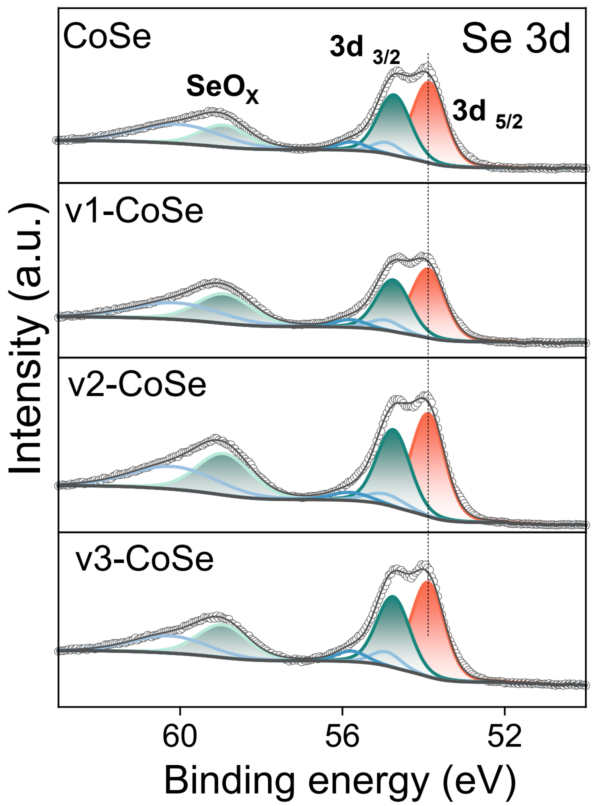

Figure S11. High-resolution Se 3d XPS spectra of CoSe and v-CoSe.

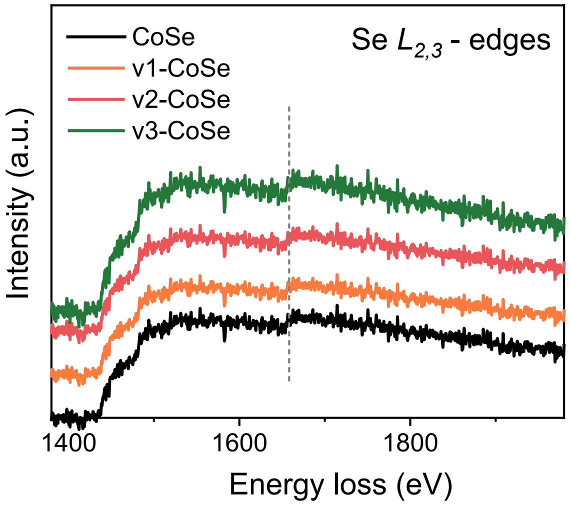

Figure S12. Se $L_{2,3}$-edges electron energy loss spectroscopy (EELS) spectra of CoSe and v-CoSe.

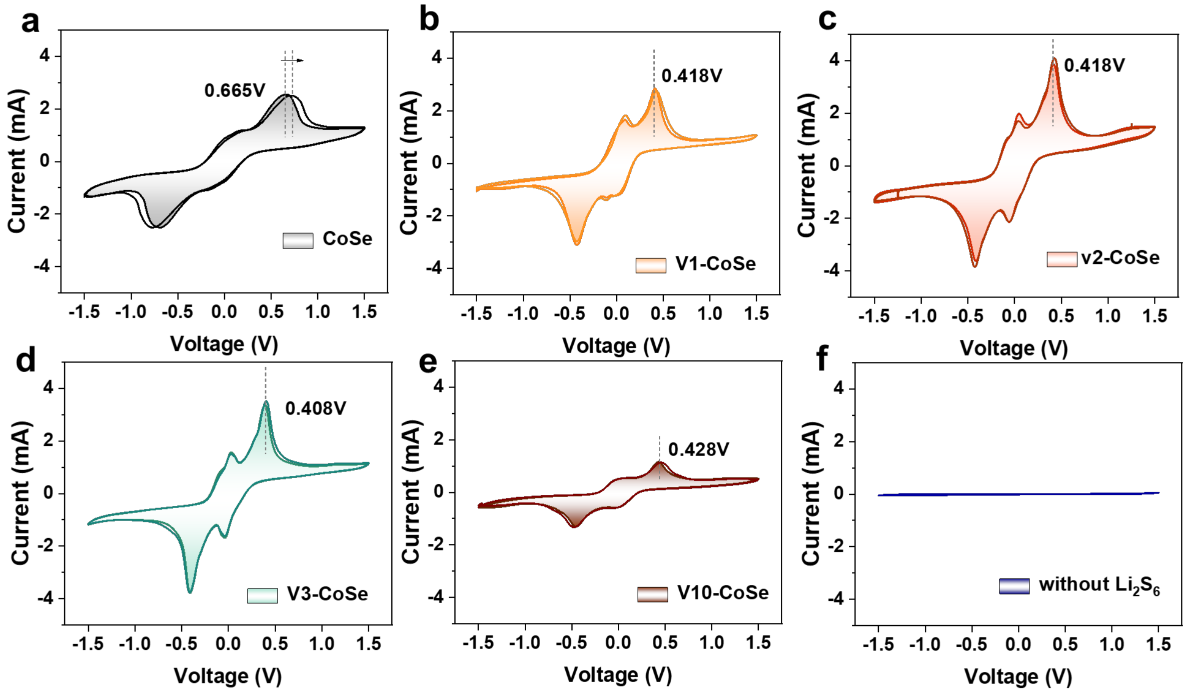

Figure S13. CV curves of symmetric cells for (a) CoSe, (b) v1-CoSe (c) v2-CoSe, (d) v3-CoSe, (e) v10-CoSe, and (f) without Li_2S_6 at a scan speed of 3 mV/s.

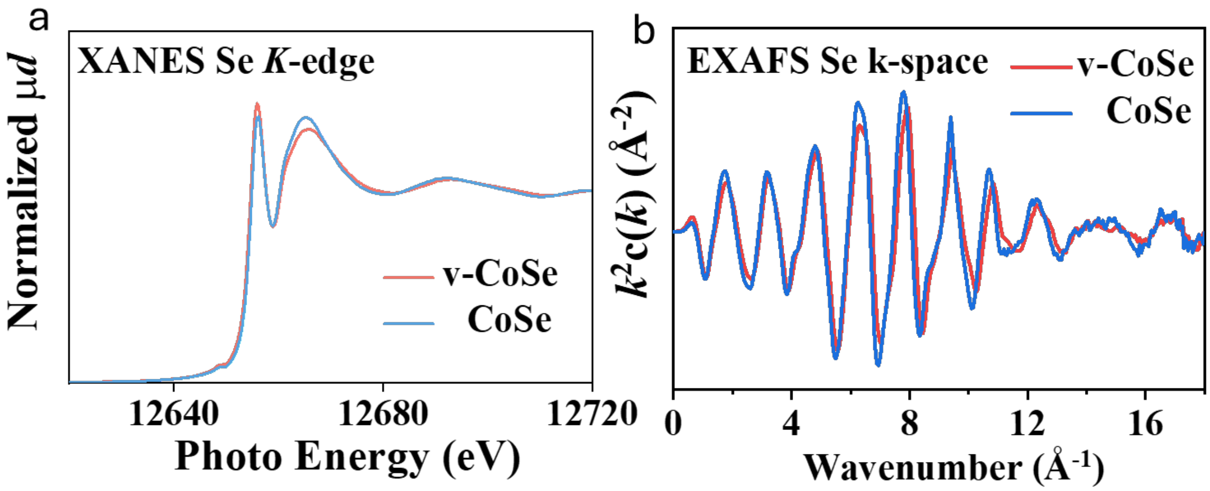

Figure S14. (a) EXANES spectra of Se K-edge. (b) EXAFS oscillation extracted from K-edge spectra of the composites in k space of CoSe and v-CoSe.

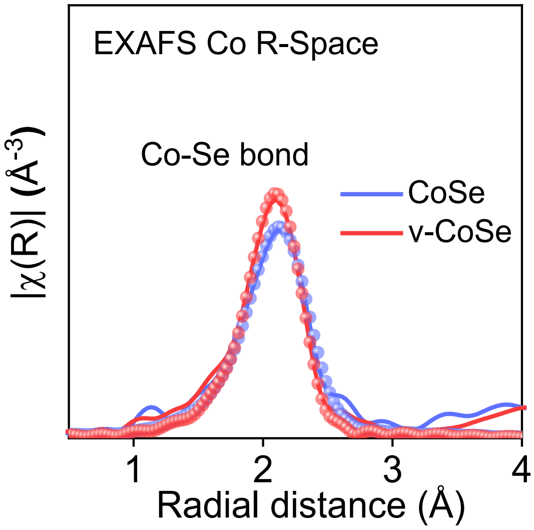

Figure S15. Co R-space EXAFS spectra of CoSe and v-CoSe.

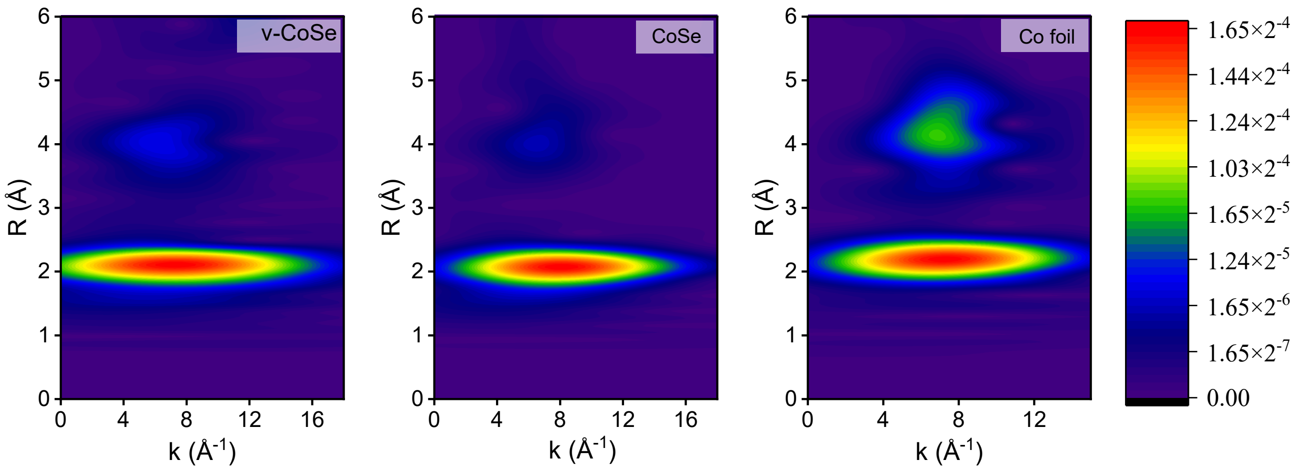

Figure S16. Wavelet-transform contour plots of the EXAFS signal of v-CoSe, CoSe, and Co foil as reference.

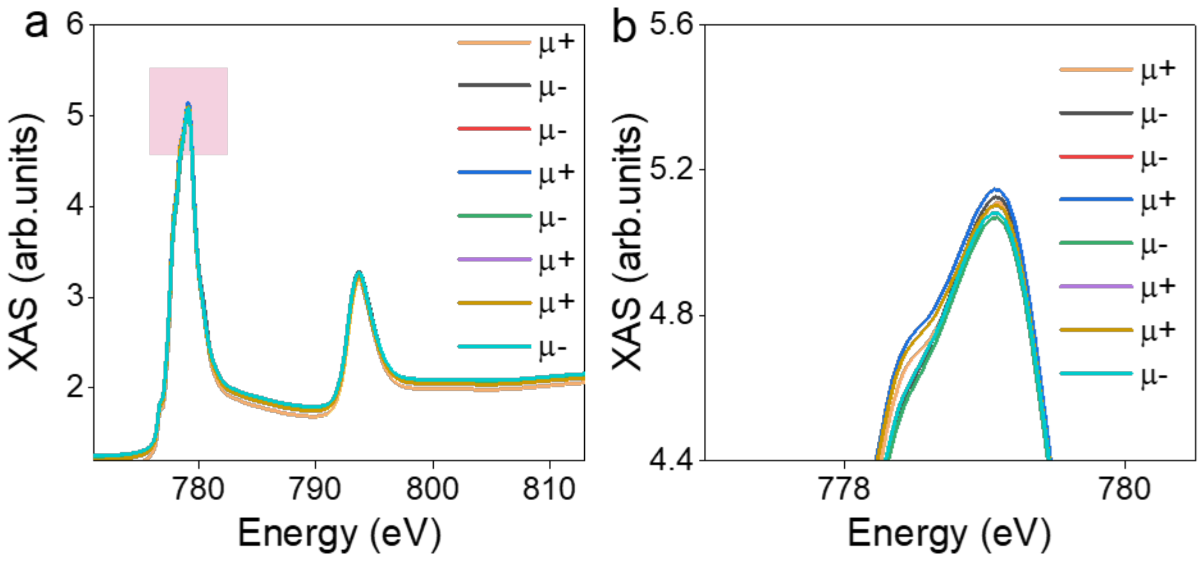

Figure S17. (a) Co $L_{2,3}$-edge X-ray absorption spectra (μ+ μ− μ− μ+ μ− μ+ μ+ μ−) of CoSe at 50 K under an applied field B = 6 T. (b) Partial enlargement of figure (a) pink region of CoSe.

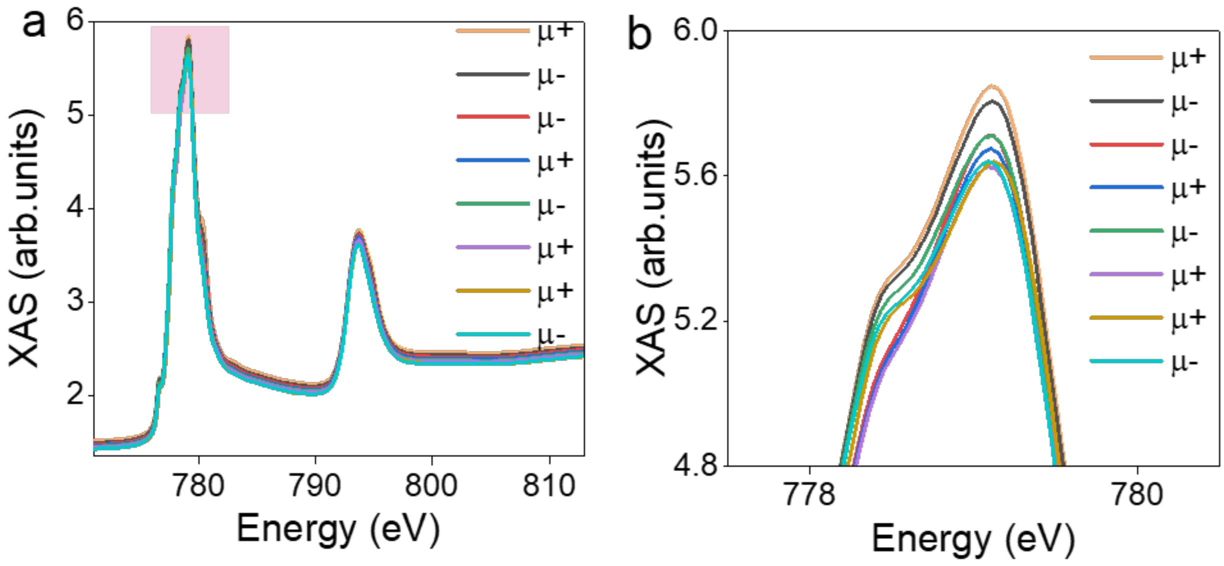

Figure S18. (a) Co $L_{2,3}$-edge X-ray absorption spectra (μ+ μ− μ− μ+ μ− μ+ μ+ μ−) of v-CoSe at 50 K under an applied field B = 6 T. (b) Partial enlargement of figure (a) pink region of v-CoSe.

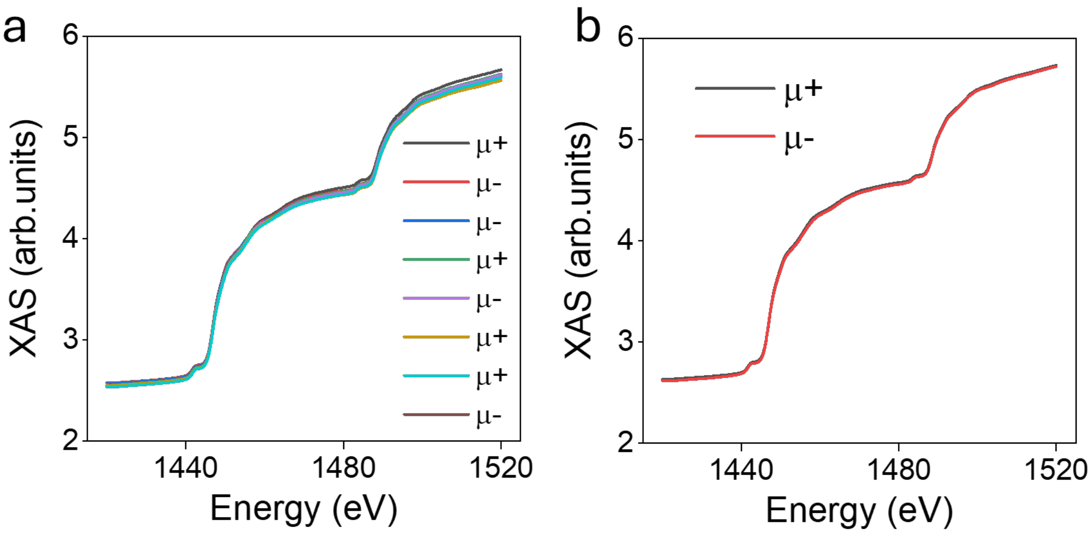

Figure S19. (a) Se L_1 edge X-ray absorption spectra (μ+ μ− μ− μ+ μ− μ+ μ+ μ−) of CoSe at 50 K under an applied field B = 6 T. (b) Average μ+ and μ− X-ray absorption spectra of CoSe.

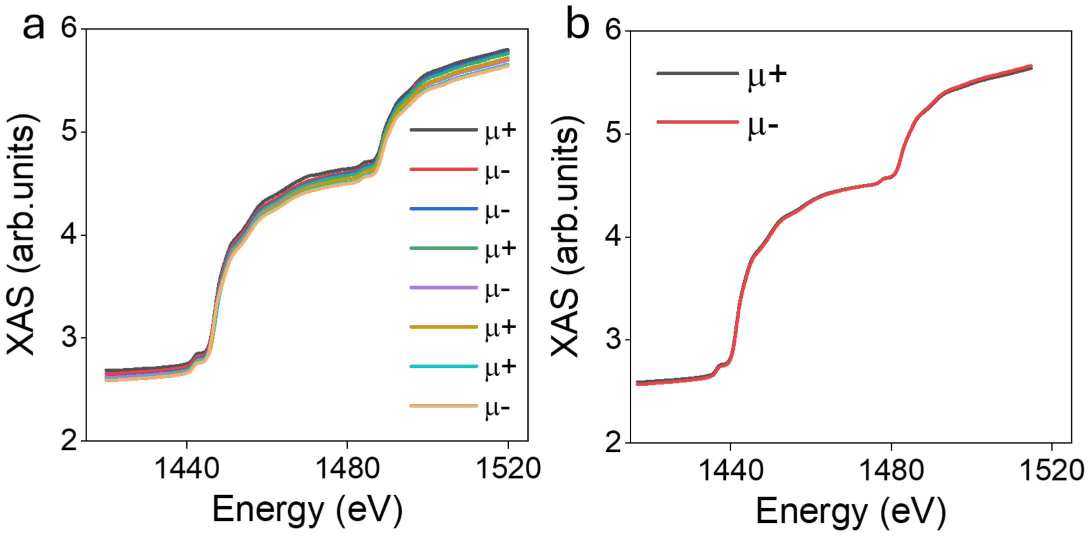

Figure S20. (a) Se L_1 edge X-ray absorption spectra (μ+ μ− μ− μ+ μ− μ+ μ+ μ−) of v-CoSe at 50 K under an applied field B = 6 T. (b) Average μ+ and μ− X-ray absorption spectra of v-CoSe.

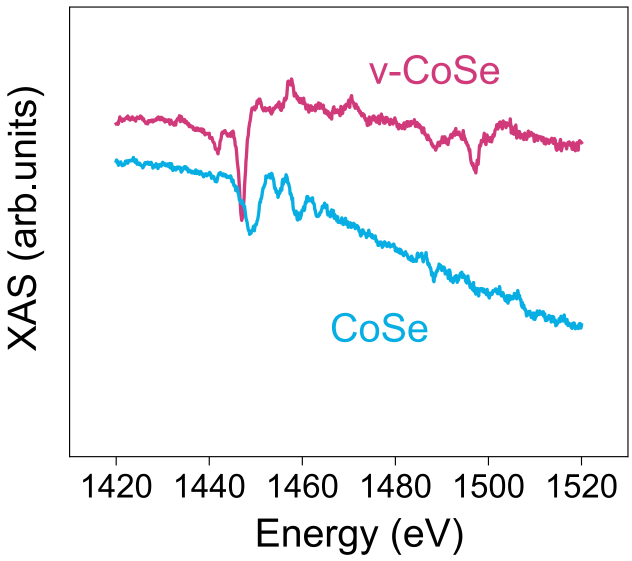

Figure S21. Se L_1 XMCD spectra of CoSe and v-CoSe at 50 K under an applied field B = 6 T.

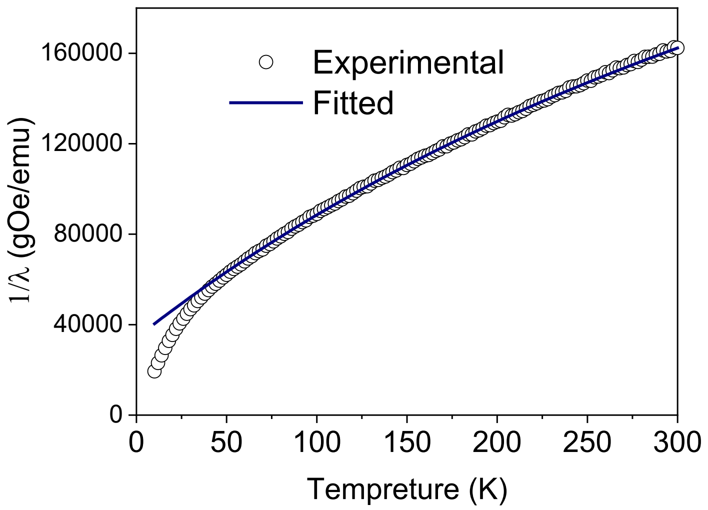

Figure S22. Temperature dependence of inverse magnetic susceptibility for v-CoSe at 50 Oe.

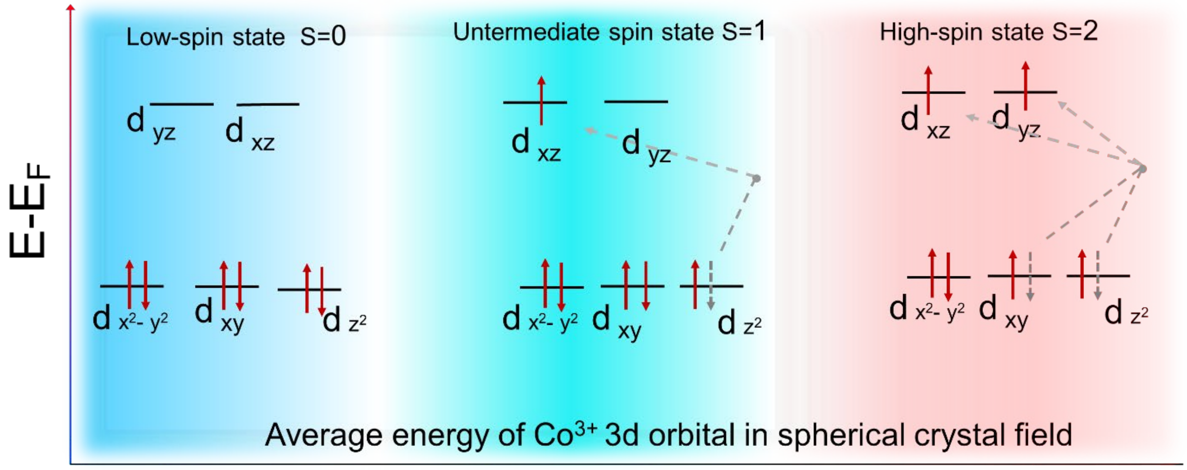

Figure S23. Schematic diagram of electron spin-orbit configuration in Co^{3+}.

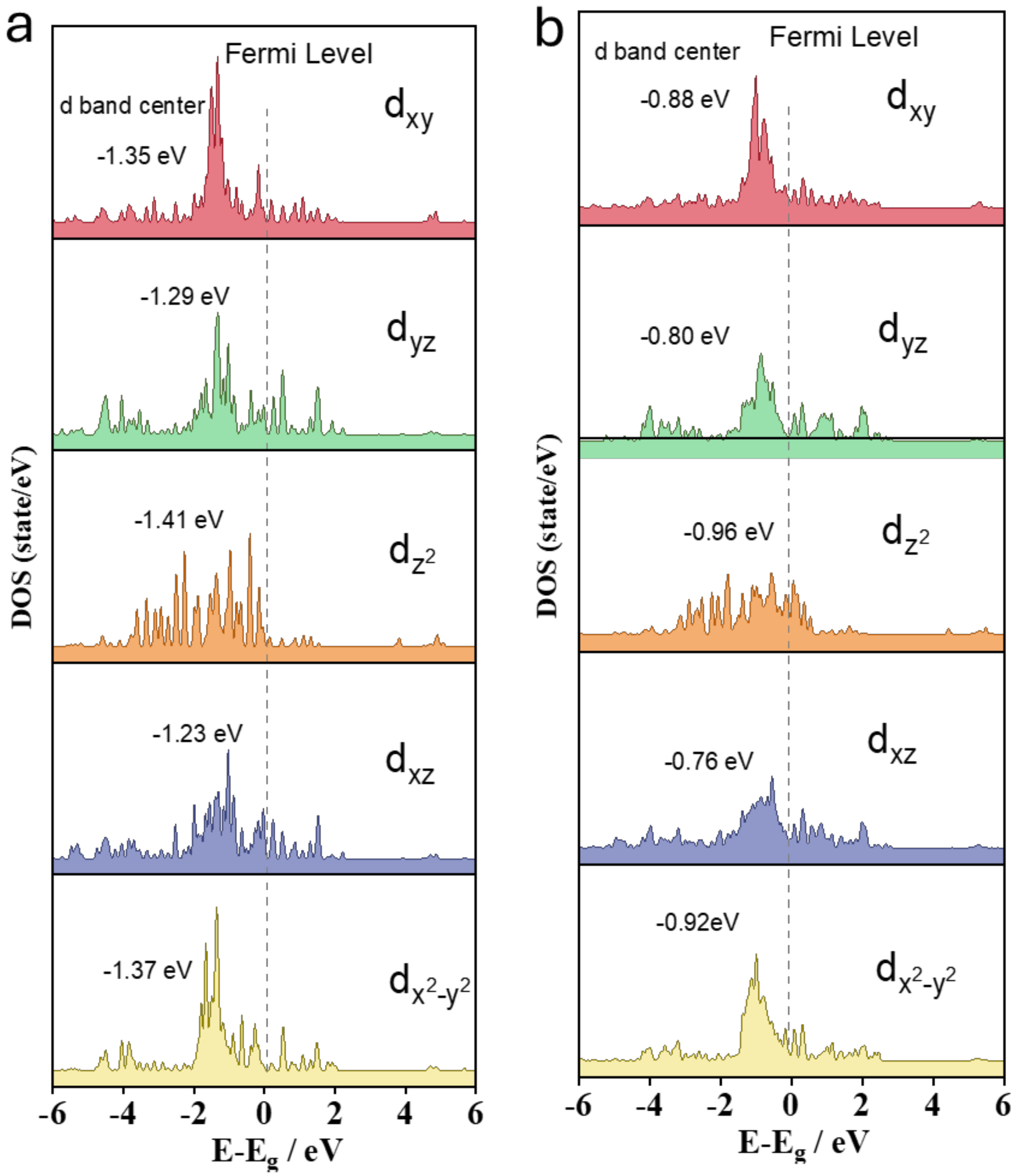

Figure S24. Partial density of states (PDOS) of Co at d-orbital for (a) CoSe and (b) v-CoSe.

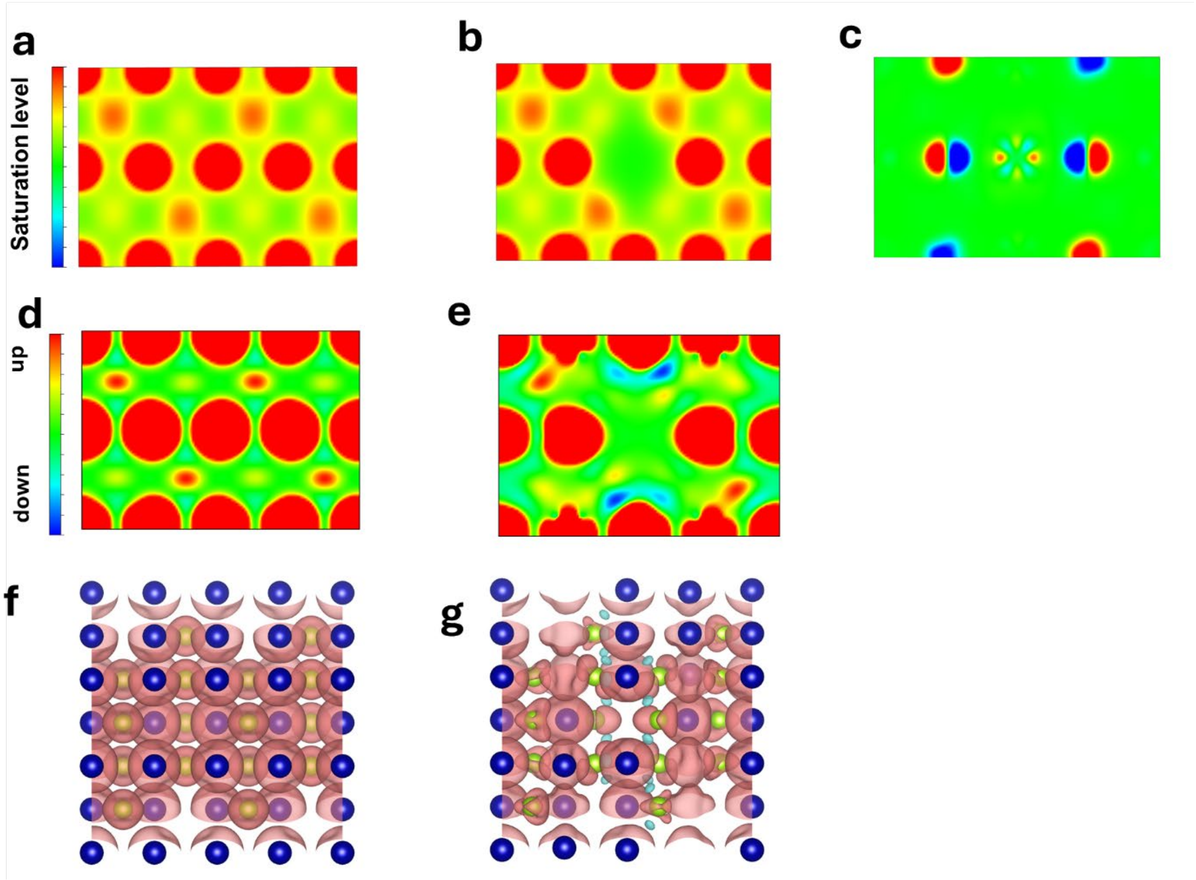

Figure S25. (a) Charge density distribution of CoSe. (b) Charge density distribution of v-CoSe. (c) Differential charge density map, between CoSe and v-CoSe. To obtain the differential map, a Co atom and its associated charge density were removed from the CoSe charge density distribution. (d) 2D spin density map of CoSe. (e) 2D spin density map of v-CoSe. (f)3D spin density model of CoSe. (g) 3D spin density model of v-CoSe.

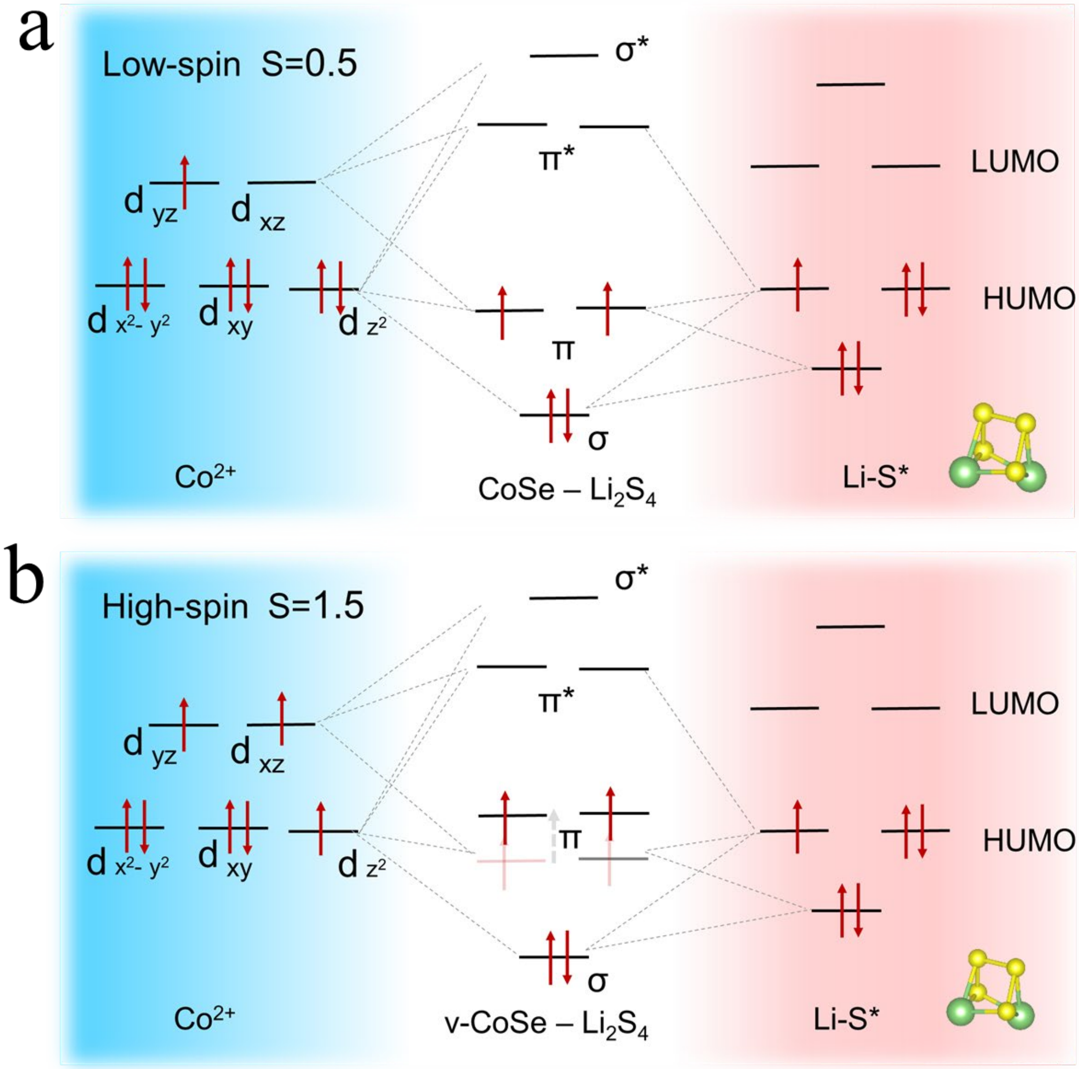

Figure S26. Illustration of Co^{2+} 3d electrons in (a) CoSe and (b) v-CoSe coupling with Li-S molecular orbital.

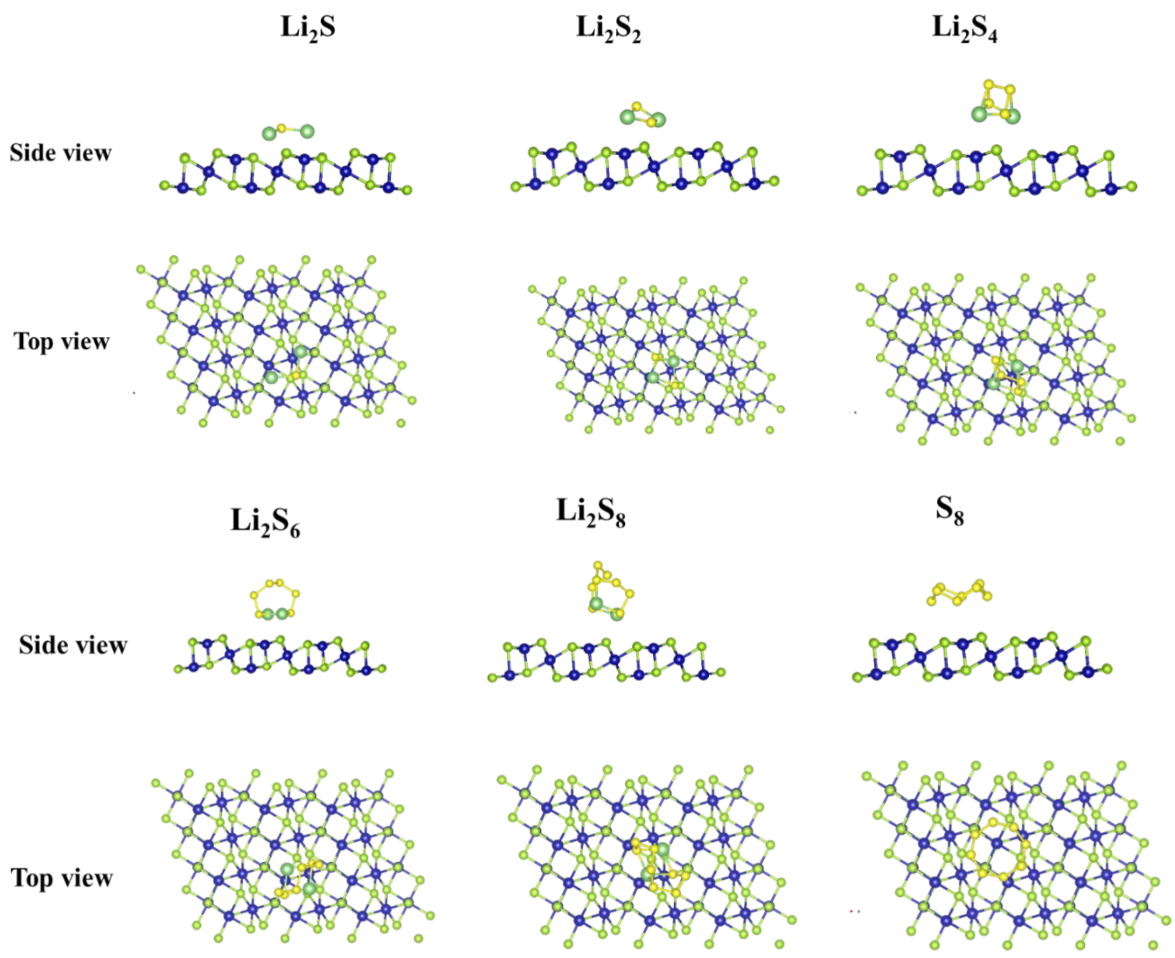

Figure S27. DFT optimized geometrical configuration of CoSe with LiPSs.

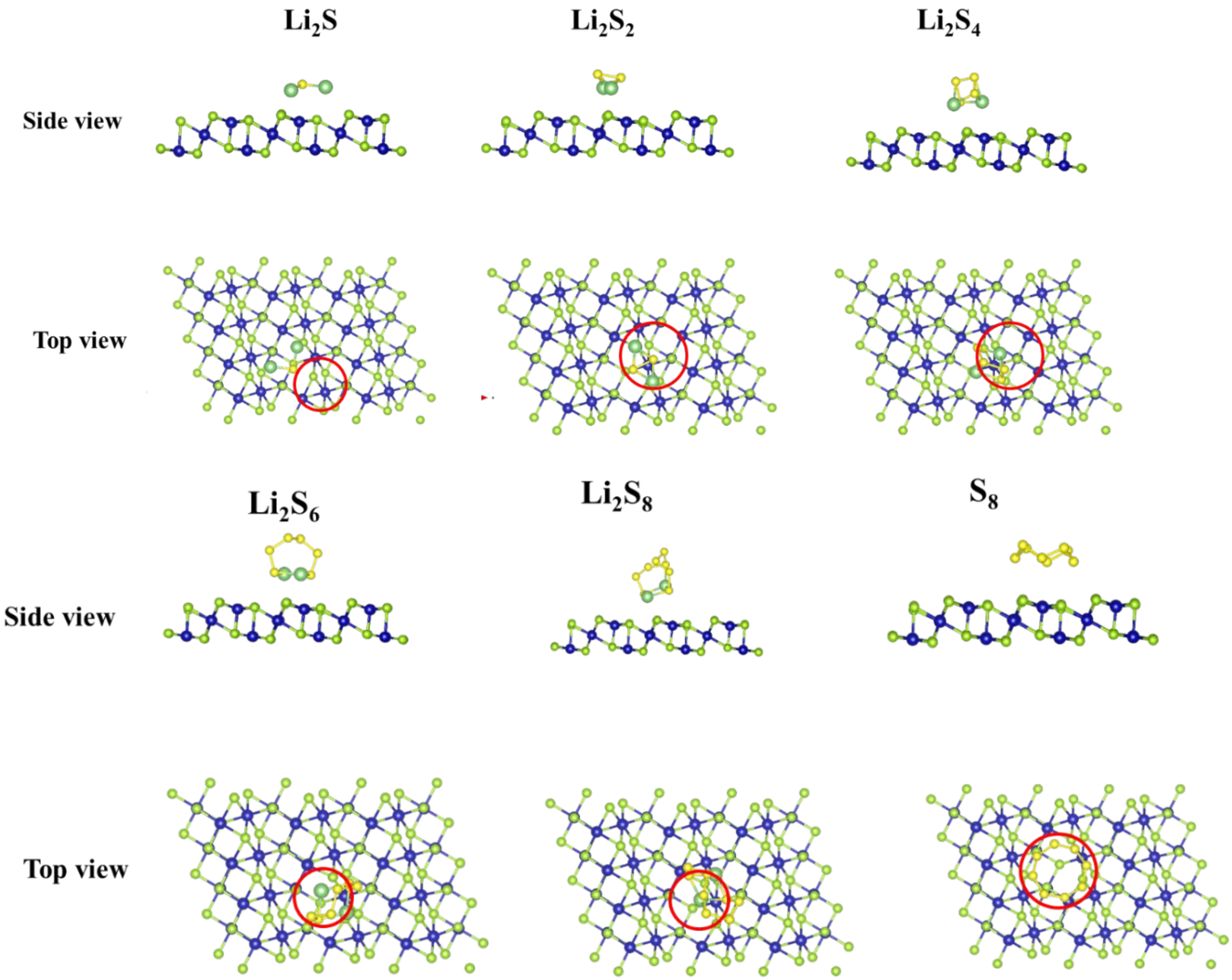

Figure S28. DFT optimized geometrical configuration of v-CoSe with LiPSs.

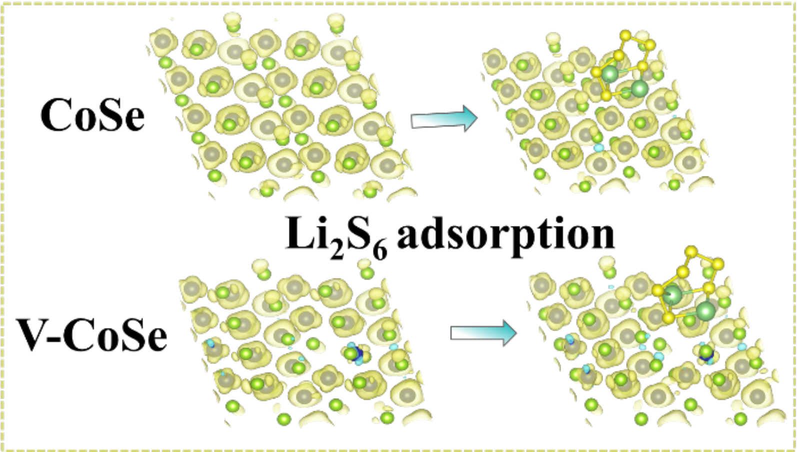

Figure 29. Top view of CoSe and v-CoSe spin density before and after Li_2S_6 adsorption.

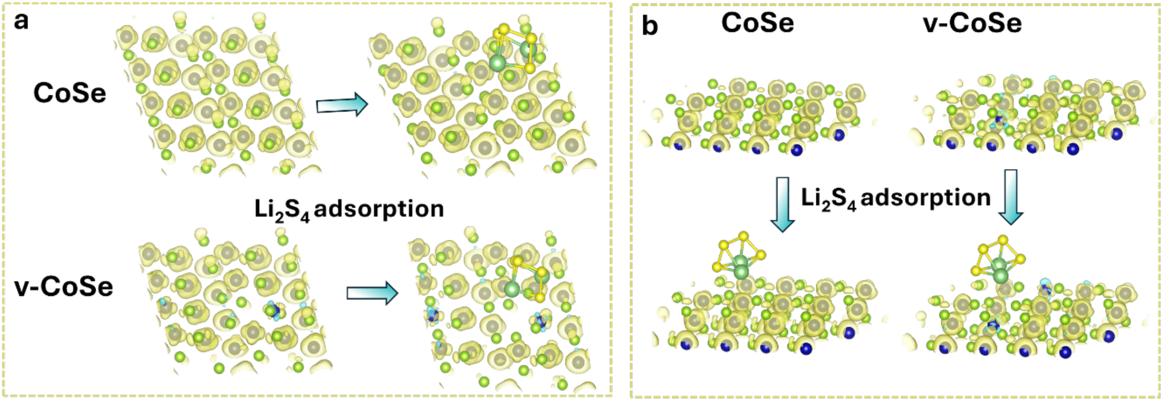

Figure 30. (a)Top view and (b) side view of the spin density of CoSe and v-CoSe before and after Li_2S_4 adsorption.

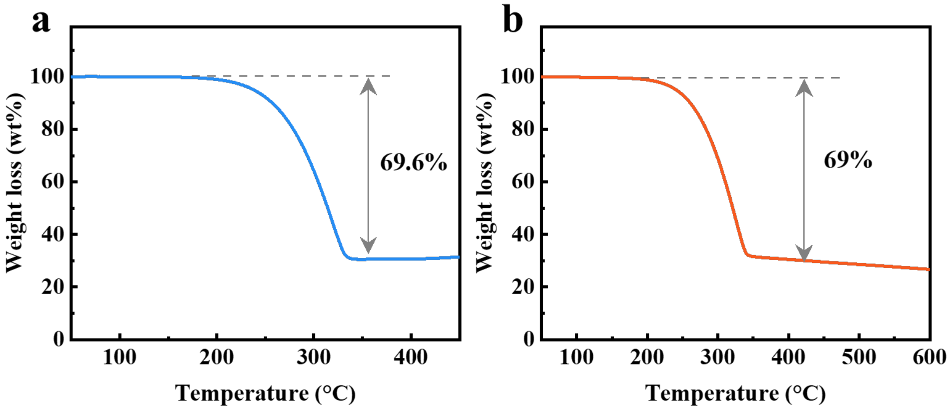

Figure S31. TGA curves of (a) CoSe and (b) v-CoSe.

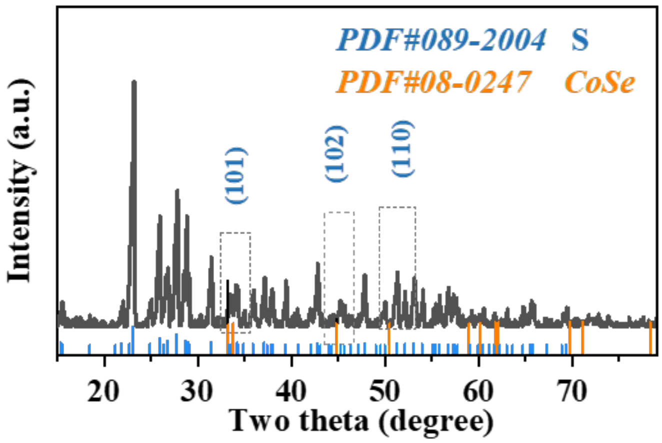

Figure S32. XRD pattern of CoSe/S.

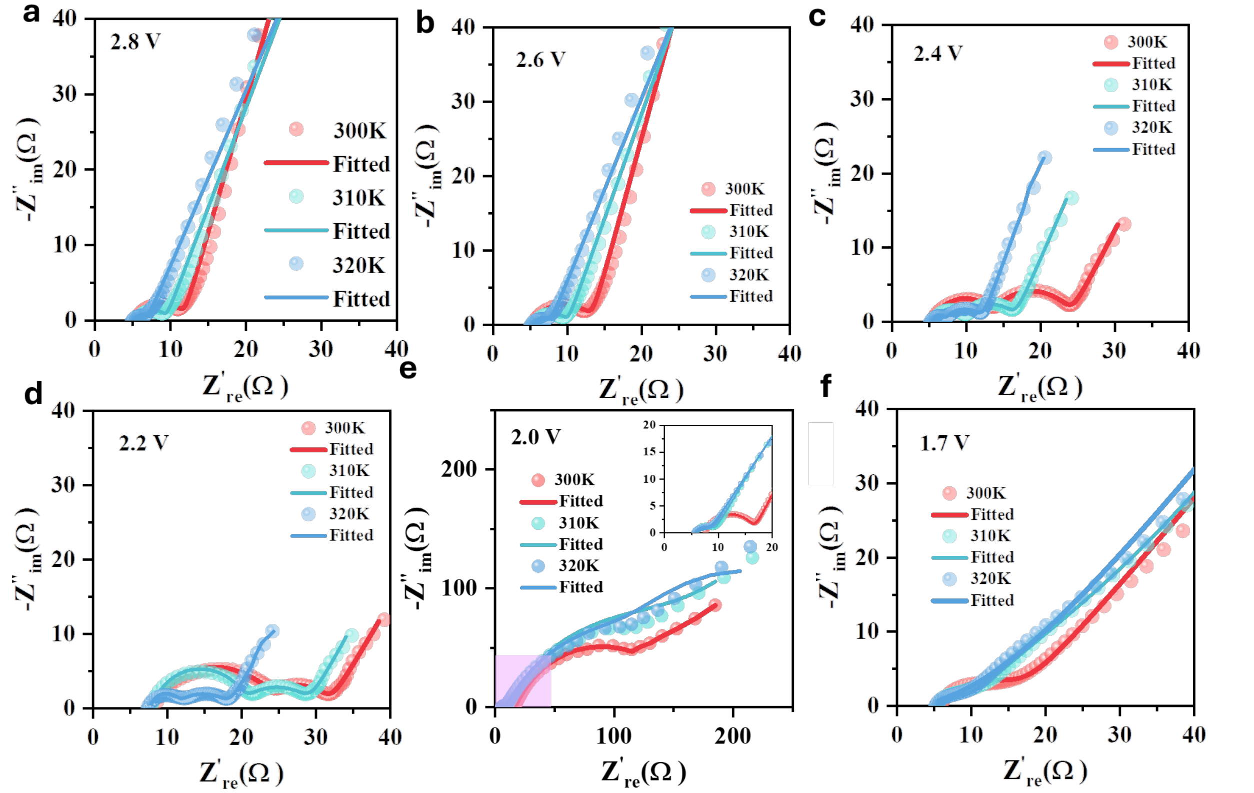

Figure S33. Nyquist plots of the EIS spectra of the CoSe/S electrodes at (a) 2.8 V, (b) 2.6 V, (c) 2.4 V, (d) 2.2 V, (e) 2.0 V with the enlarged figure inserted, and (f) 1.7 V during discharging at different temperatures (300 K, 310 K, and 320 K). The raw impedance data and the fitted data are shown as symbols and lines, respectively.

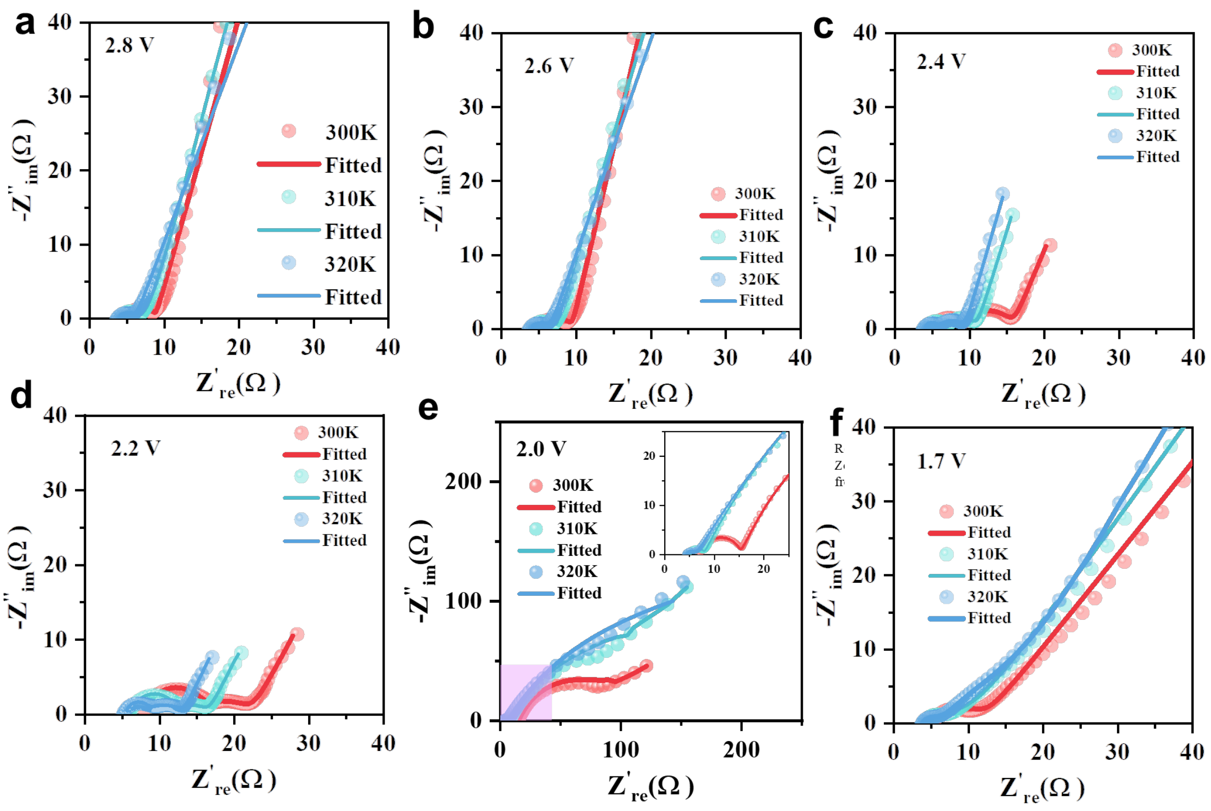

Figure S34. Nyquist plots of the EIS spectra of the v-CoSe/S electrodes at (a) 2.8 V, (b) 2.6 V, (c) 2.4 V, (d) 2.2 V, (e) 2.0 V with the enlarged figure inserted, and (f) 1.7 V during discharging at different temperatures (300 K, 310 K, and 320 K). The raw impedance data and the fitted data are shown as symbols and lines, respectively.

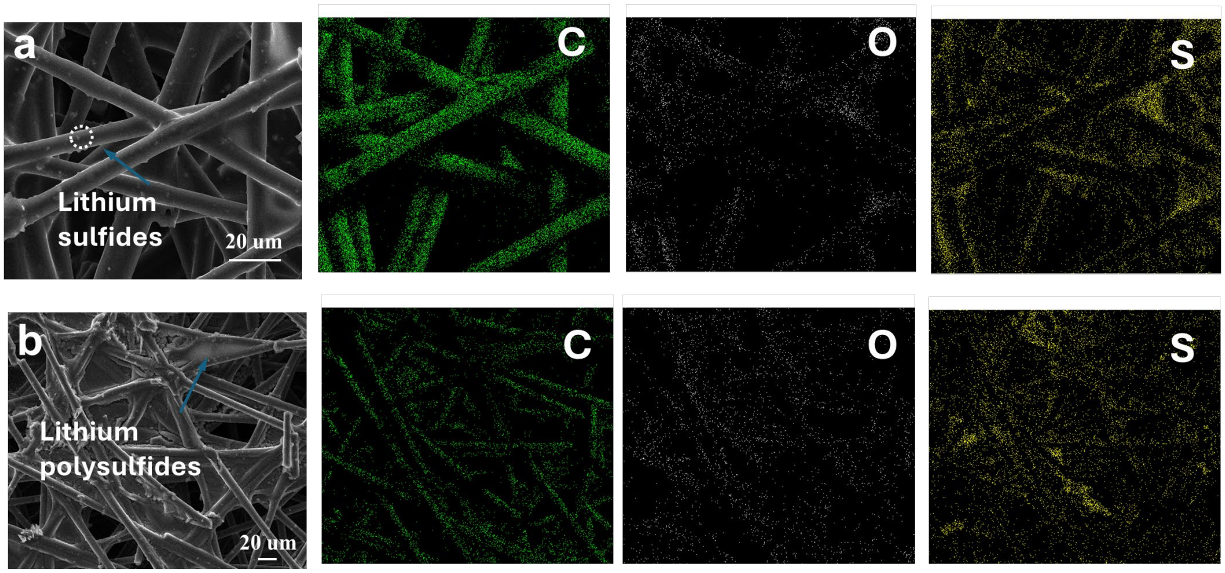

Figure S35. SEM images and EDX elemental maps of lithium sulfide particles deposited on a carbon paper electrode of (a) CoSe/S and (b) v-CoSe/S.

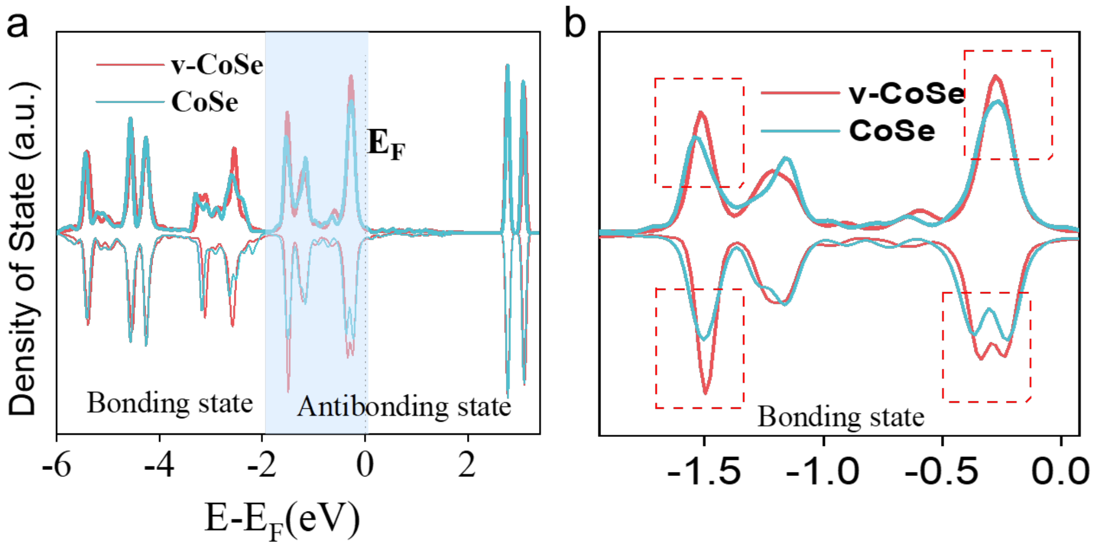

Figure S36. (a) PDOS of bonding state orbital hybridization for Li_2S_4 of CoSe and v-CoSe, (b) Enlarged view of the blue region of the panel (a).

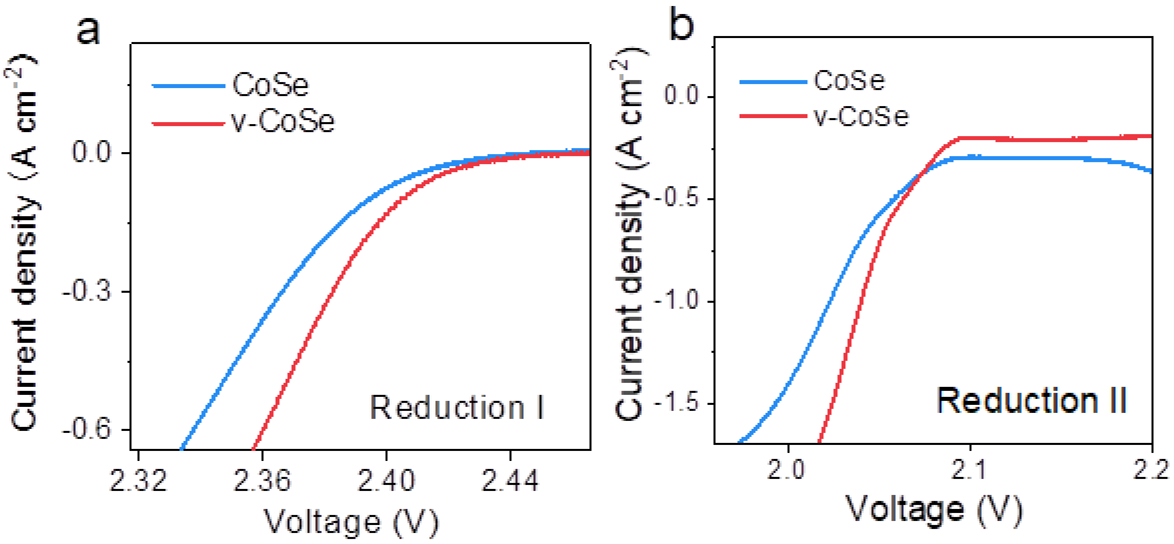

Figure S37. Linear sweep voltammetry (LSV) curves of (a) reduction I and (b) reduction II processes from the Figure 5a pink regions.

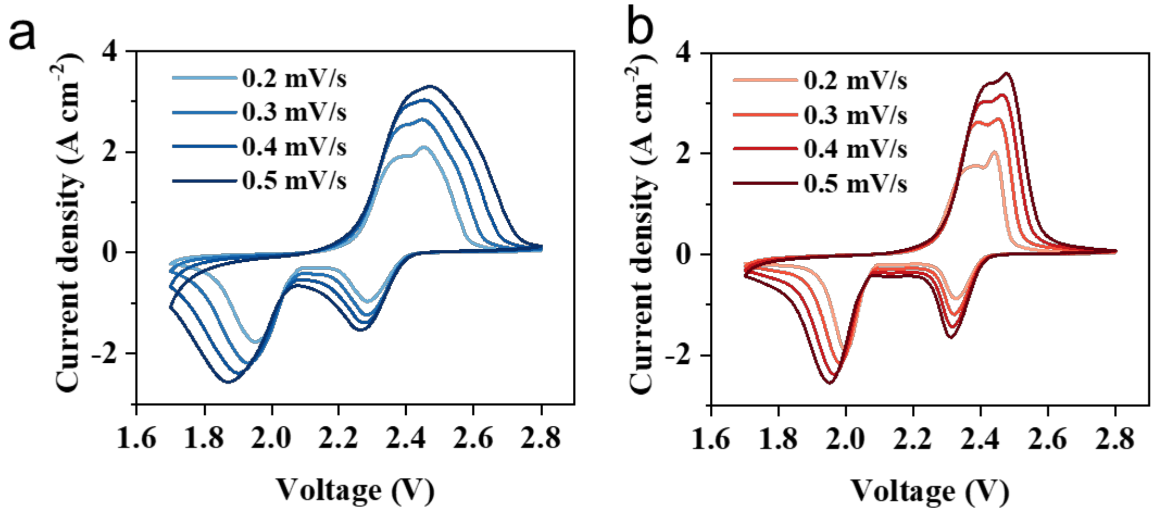

Figure S38. CV curves of (a) CoS/S and (b) v-CoSe/S at different scanning rates.

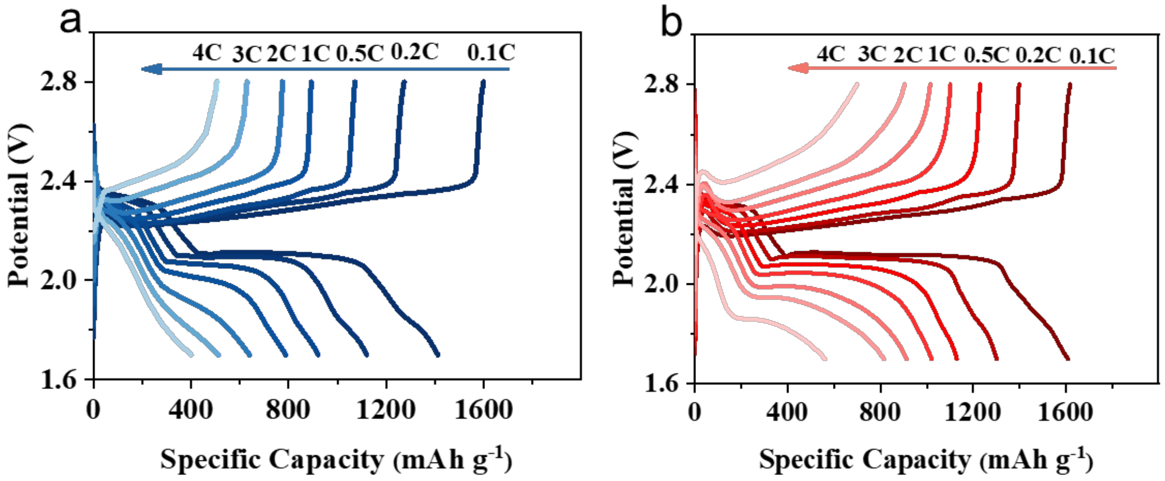

Figure S39. Galvanostatic charge and discharge curves of (a) CoSe/S and (b) v-CoSe/S at different current densities.

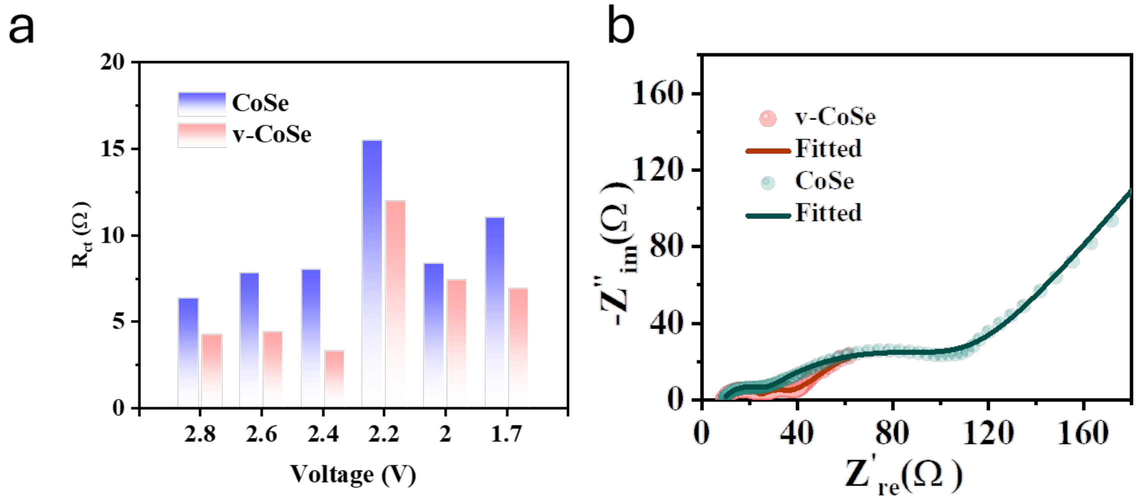

Figure S40. (a) Fitted R_{ct} values at different discharge voltage during the first discharge process at 300 K. (b) EIS spectra and fitting results after 1500 cycles of CoSe and v-CoSe.

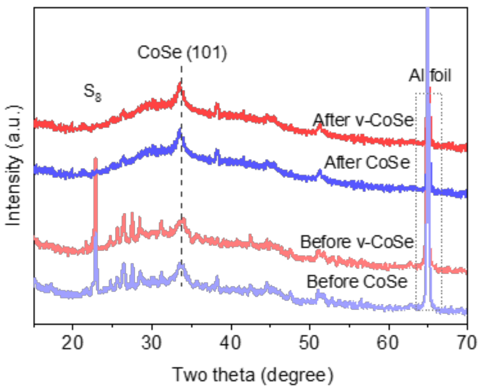

Figure S41. XRD patterns of CoSe/S and v-CoSe/S electrodes before and after cycling.

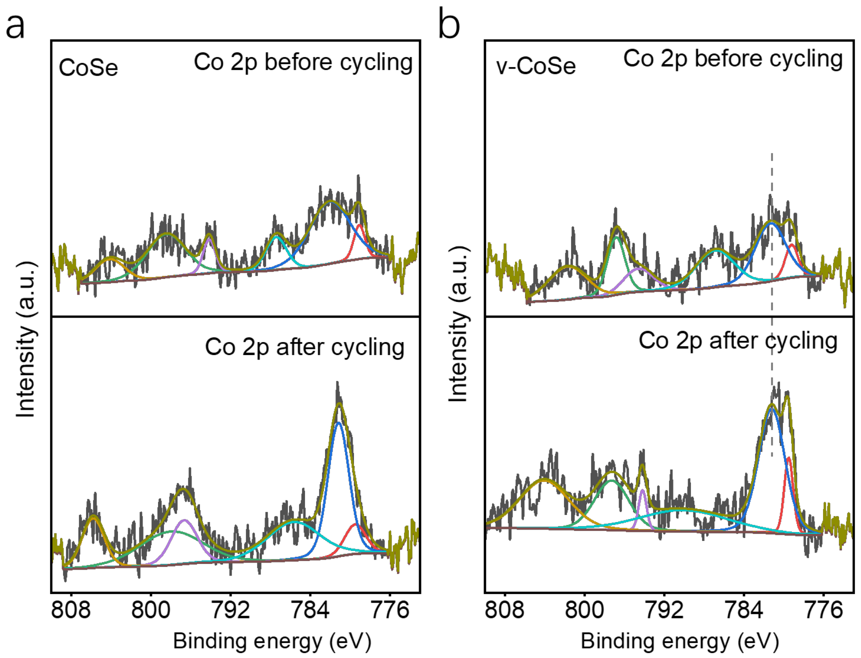

Figure S42. High-resolution Co 2p XPS spectra of CoSe/S and v-CoSe/S electrodes before and after cycling.

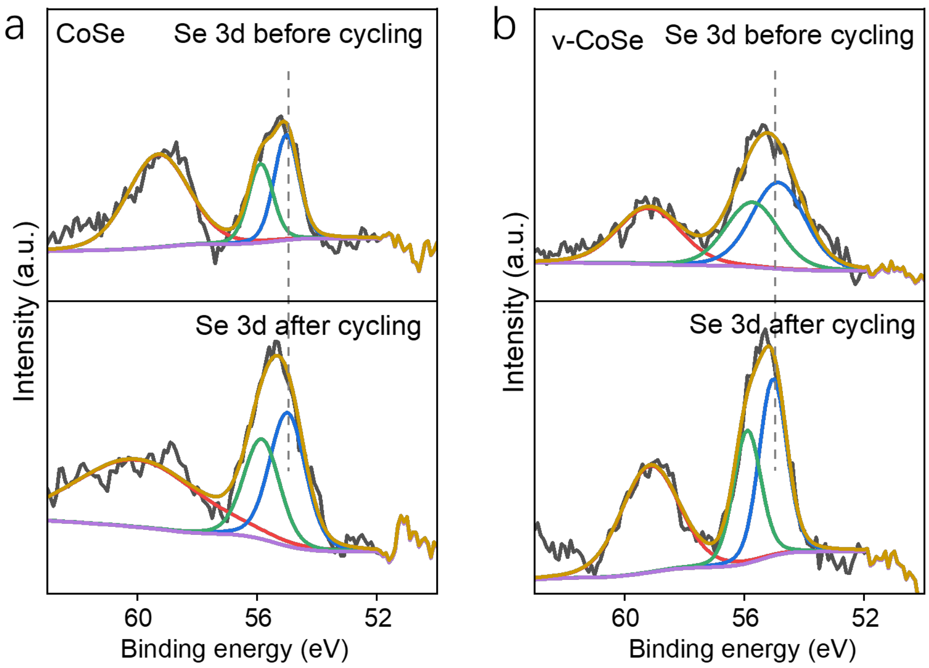

Figure S43. High-resolution Se 3d XPS spectra of CoSe/S and v-CoSe/S electrodes before and after cycling.

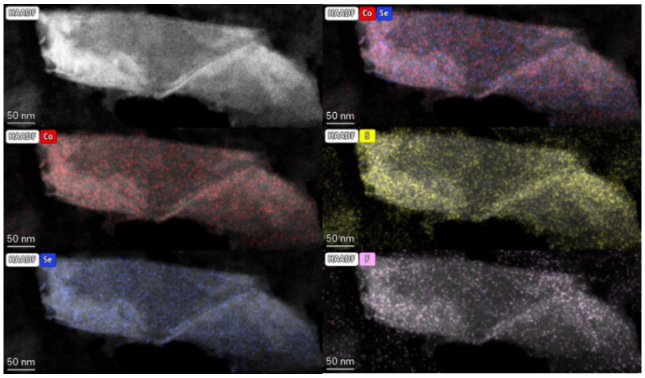

Figure S44. EDS of CoSe after 1000 charge - discharge process.

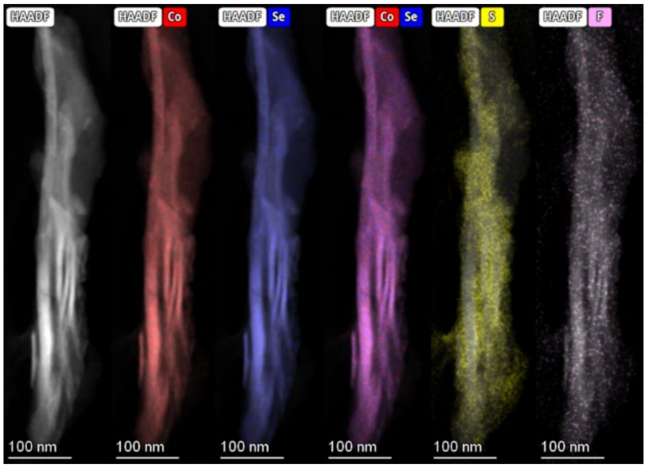

Figure S45. EDS of v-CoSe after 1000 charge - discharge process.

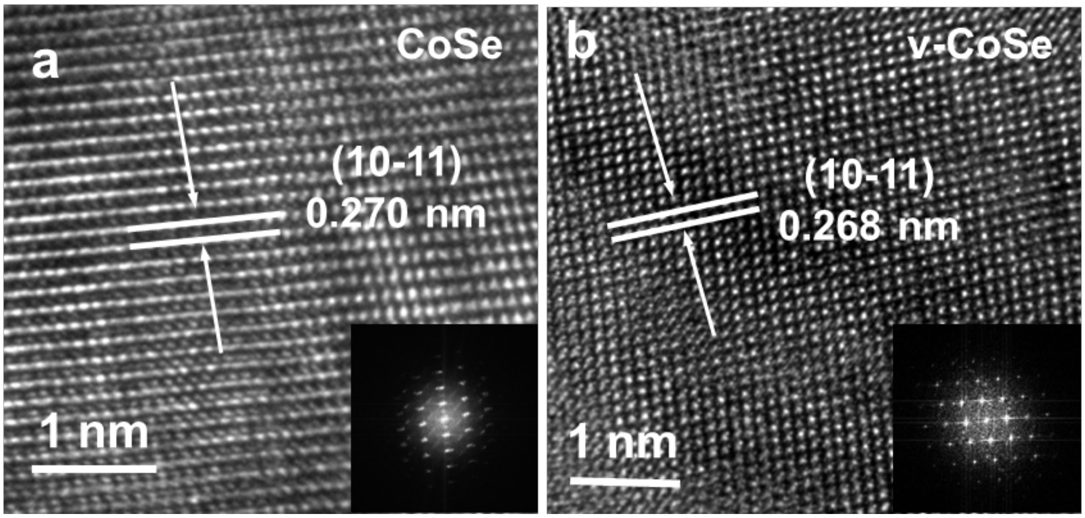

Figure S46. HRTEM of (a) CoSe/S and (b) v-CoSe/S electrodes after cycling.

Table S2. AC-HAADF-STEM index of CoSe and v-CoSe.

Sample	Spot	Experimental (nm)	CoSe (hexagonal) [0001]
CoSe	1	0.181	0.181 (11-20)
	2	0.180 (60.00º vs Spot 1)	0.181 (60.00º vs Spot 1) (2-1-10)
	3	0.103 (30.00º vs Spot 1)	0.105 (30.00º vs Spot 1) (30-30)
v-CoSe	1	0.179	0.181 (11-20)
	2	0.179 (60.00º vs Spot 1)	0.181 (60.00º vs Spot 1) (2-1-10)
	3	0.102 (30.00º vs Spot 1)	0.105 (30.00º vs Spot 1) (30-30)

Table S3. Atomic fraction from EDS spectra of CoSe and v-CoSe.

Catalysts	Element	Atomic Fraction (%)	Atomic Error (%)
CoSe	Co	52.40	3.5
	Se	47.60	3.5
v1-CoSe	Co	45.10	3.6
	Se	54.90	3.6
v2-CoSe	Co	44.30	3.5
	Se	55.70	3.5
v3-CoSe	Co	43.18	3.5
	Se	56.82	3.5

Table S4. Overpotential and square area of symmetrical cells CV curves of different catalysts.

Catalyst	Charge (Coulomb)	Overpotential
CoSe	3.11	0.665 V
v1-CoSe	2.45	0.418 V
v2-CoSe	3.53	0.418 V
v3-CoSe	2.78	0.408 V
v10- CoSe	1.44	0.428 V
Without	0.05	——

Table S5. Structural parameters extracted from the Se K-edge EXAFS fitting.

Sample	Bonding	R (Å)	R error (Å)	N	N error	σ^2
CoSe	Se-Co	2.4048	0.0049	4.2	(S_0^2 = 0.286)	0.0074
v-CoSe	Se-Co	2.4297	0.0064	5.0	(S_0^2 = 0.445)	0.0075

Table S6. Structural parameters extracted from the Co K-edge EXAFS fitting.

Sample	Bonding	R (Å)	R error (Å)	N	N error	σ^2
Co foil	Co-Co	2.4978	0.0057	12	(S_0^2 = 0.86)	0.0071
CoSe	Co-Se	2.42546	0.0093	4.8	(S_0^2 = 0.73)	0.0082
	Co-Co	2.63225	0.0376	1.2	(S_0^2 = 0.422)	0.0082
v-CoSe	Co-Se	2.4119	0.0061	5.4	(S_0^2 = 0.679)	0.0082
	Co-Co	2.67785	0.0230	1.0	(S_0^2 = 0.421)	0.0082

Table S7. Adsorption energy of polysulfides by CoSe and v-CoSe.

**Sample**	Li_2S	Li_2S_2	Li_2S_4	Li_2S_6	Li_2S_8	S_8
CoSe	1.62 eV	-1.06eV	-0.21 eV	-0.13 eV	-0.17 eV	-0.0089 eV
v-CoSe	-1.63 eV	-1.08 eV	-0.22 eV	-0.19 eV	-0.25 eV	-0.12 eV
Adsorption energy increase percentage (%)	0.6	1.9	4.7	46.1	47.1	

Reference

[1] Arbiol, J.; Cirera, A.; Peiró, F.; Cornet, A.; Morante, J. R.; Delgado, J. J.; Calvino, J. J. Optimization of tin oxide nanosticks faceting for the improvement of palladium nanoclusters epitaxy. *Appl. Phys. Lett.* **2002**, *80*, 329–331.

[2] Bernal, S.; Botana, F.; Calvino, J.; Lopez-Cartes, C.; Perez-Omil, J.; Rodriguez-Izquierdo, J. The interpretation of HREM images of supported metal catalysts using image simulation: profile view images. *Ultramicroscopy*, **1998**, *72*, 135–164.

[3] Grillo, V.; Rotunno, E. STEM_CELL: A software tool for electron microscopy: Part I—simulations. *Ultramicroscopy*, **2013**, *125*, 97–111.

[4] Malis, T.; Cheng, S. C.; Egerton, R.F. EELS log-ratio technique for specimen-thickness measurement in the TEM. *Journal of Electron Microscopy Technique*. **1988**, *8*, 193–200.

[5] Liu, Y.; Cheng, H.; Lyu, M.; Fan, S.; Liu, Q.; Zhang, W.; Zhi, Y.; Wang, C.; Xiao, C.; Wei, S.; Ye, B.; Xie, Y. Low Overpotential in Vacancy-Rich Ultrathin CoSe2 Nanosheets for Water Oxidation, *J. Am. Chem. Soc.* **2014**, *136*, 15670–15675.
